# Ecological and functional roles of plant microbiomes in environmental detoxification

**DOI:** 10.3389/fmicb.2026.1883316

**Published:** 2026-07-15

**Authors:** Samy Selim, Krishnendu Adhikary, Riya Sarkar, Krishnendu Ganguly, Arijit Misra, Alaa A. Kashmiry, Sahar A. Alshareef, Shaza N. Alkhatib, Nashwa Hagagy, Rajkumar Maiti

**Affiliations:** 1 Department of Clinical Laboratory Sciences, College of Applied Medical Sciences, Jouf University, Sakaka, Saudi Arabia; 2 Department of Medical Laboratory Technology, Paramedical College Durgapur, Durgapur, India; 3 Research and Development Cell, Lovely Professional University, Jalandhar, Phagwara, India; 4 Department of Medical Laboratory Technology, Dr. B. C. Roy Academy of Professional Courses, Fuljhore, Durgapur, India; 5 Department of Chemistry, Applied College at Khulais, University of Jeddah, Jeddah, Saudi Arabia; 6 Department of Biological Sciences, College of Science, University of Jeddah, Jeddah, Saudi Arabia; 7 Department of Biological Sciences, College of Science and Arts at Khulais, University of Jeddah, Jeddah, Saudi Arabia; 8 Department of Botany and Microbiology, Faculty of Science, Suez Canal University, Ismailia, Egypt; 9 Department of Physiology, Bankura Christian College, Bankura, India

**Keywords:** environmental detoxification, microbial functional ecology, plant microbiome, rhizosphere interactions, sustainable bioremediation

## Abstract

Plant-associated microbiomes play a crucial role in environmental detoxification by influencing the degradation, immobilization, and resistance to toxins in polluted settings. The ecological and functional activity of endogenous microbial communities, such as rhizobacteria and endophytic microorganisms, is not well studied when examining contaminants and their environments, despite the fact that plant-mediated bioremediation has garnered a lot of research attention. The majority of previously published research focuses on a single biodegradation route or solitary plant-microbe interactions. Our knowledge of how the microbiome’s composition, functional diversity, and ecological stability of microbial communities work together to produce detoxifying results in practical applications is currently lacking. To advance understanding of how plant microbiomes cooperatively mediate environmental detoxification through metabolic interactions, adaptive responses, and host–microbiome communication, this review integrates insights from microbial ecology and functional microbiology. Its primary objective is to synthesize current knowledge on key microbial functions, including metal sequestration, xenobiotic degradation, redox regulation, and modulation of plant responses to biotic stress, while linking these functions to ecological processes such as host specificity, niche specialization, and community assembly. A distinctive aspect of this review is its ecosystem-level perspective, which shifts the focus from individual microbial taxa to the functional resilience of microbial communities in determining detoxification efficiency. The information provided in this review has a scope to provide framework to develop ecologically-sustaining, microbiome-based strategies for the detoxification of the environment and for conducting future bioremediation research.

## Graphical abstract

**Figure:**
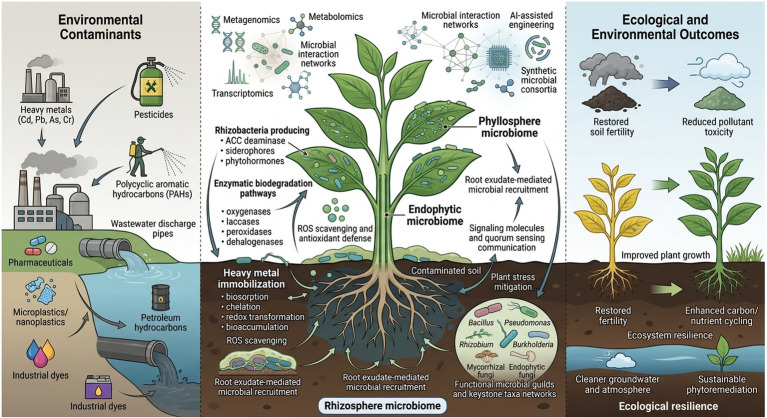

## 1 Introduction

Environmental contamination in terrestrial and aquatic ecosystems has been accelerated by industrialization, urbanization, intensive agriculture and unsustainable waste disposal (Rai et al., 2021). Toxic metals, petroleum hydrocarbons, pesticides, pharmaceuticals, plastics and persistent organic pollutants are now ubiquitous in soils, sediments and water bodies, altering ecosystem function at local and global scales (Jiang et al., 2020). Many of these contaminants are chemically stable and bioaccumulative and can enter food webs, and their long-term accumulation has become a major ecological and public health concern. Such pollutants affect microbial diversity, plant productivity, animal health and human populations through direct toxicity and indirect disruption of the ecosystem (Prata et al., 2020). Moreover, the complexity of contamination is compounded by mixed-pollutant scenarios where multiple compounds interact synergistically, making predictions and remediations increasingly difficult. In this context, understanding natural biological systems that can withstand and transform environmental pollutants has emerged as a critical scientific priority (Vasse and Melgert, 2024).

Plants are the main interface between soil, atmosphere and associated microbial communities and are therefore central to contaminated landscapes (Symeonides et al., 2024). Their roots are constantly in contact with surrounding microorganisms, creating highly dynamic microhabitats that affect nutrient turnover, chemical transformation and ecological resilience (Meng et al., 2025). During the last decade, research has increasingly shown that plants are not the only actors of environmental adaptation, but they are rather holobionts in which host tissues and associated microbiomes act as integrated biological systems (Rai et al., 2022). This concept has transformed environmental biotechnology by demonstrating that tolerance and detoxification of pollutants is often a function of coordinated plant-microbe interactions rather than isolated plant physiology. Root-associated bacteria, endophytes and rhizosphere fungi are involved in the degradation, immobilization or transformation of xenobiotic compounds, often improving plant survival under toxic stress (Agrawal et al., 2024).

A conceptual change in environmental remediation science has occurred due to increased awareness of plant microbiomes (Chang et al., 2020). The focus is shifting not only to engineered detoxification technologies, but to biological partnerships that evolved naturally in contaminated habitats. Plant-associated microbial consortia not only improve detoxification efficiency but also ensure ecosystem recovery by restoring nutrient cycling and stabilizing soil structure. This ecological view is especially important in the current climate change scenario, where contamination is combined with drought and salinity and temperature stress (Le et al., 2024). Thus, investigating plant microbiomes provides a promising framework for integrating ecology, microbiology, and environmental engineering toward sustainable remediation strategies.

In this regard, the current review covers the ecological and functional significance of plant microbiomes in environmental detoxification (Kumar et al., 2024). The rationale is to bring together the sparse data available on microbial-assisted pollutant transformation and to acknowledge how host plants control such processes in the face of changing environmental conditions. The main goal is to synthesize current knowledge on microbiome-mediated detoxification mechanisms, while highlighting ecological interactions that drive resilience of pollutants. The aims are to assess the limitations of conventional remediation, to outline the rise of plant microbiomes as detoxifying agents, to examine the functional microbial processes in polluted ecosystems and to identify future directions for microbiome-based environmental restoration.

### 1.1 Environmental contamination and limitations of conventional remediation

Environmental contamination has spread from isolated industrial sites to agricultural lands, urban peripheries, wetlands, rivers, and even to remote ecosystems (Sridhar and Parimalarenganayaki, 2025). Mining, industrial discharge and fertilizer use result in heavy metals like cadmium, arsenic, chromium and lead persisting in soils, while synthetic chemicals such as herbicides, pharmaceuticals, dyes and petroleum derivatives accumulate due to anthropogenic activities. Many of these contaminants remain in the environment for long periods of time and can interfere with microbial metabolism, nutrient availability and trophic interactions, disrupting biochemical cycles (Belmaker et al., 2024). They tend to be persistent and have led to chronic exposure scenarios where damage to the ecology is slow but widespread. The magnitude of this pollution has increased the need for remediation technologies that can deal with heterogeneous pollutant loads with minimum disturbance to the ecology (Sohail et al., 2023).

Conventional remediation techniques to alleviate the pollutant burden, such as excavation, thermal treatment, chemical oxidation, soil washing, and physical containment, have been widely used. These technologies typically provide an immediate reduction in contaminant concentration, but are associated with high operational costs, energy needs, and secondary environmental impacts (Okoffo et al., 2022). Mechanical excavation can disrupt the soil architecture and often does not remove pollution but moves it to disposal sites. Chemical treatments can generate toxic by-products, change soil chemistry and negatively impact native microbial communities crucial for ecosystem recovery. Similarly, sterilizing soils through thermal treatments results in loss of fertility and long restoration times. These drawbacks are especially apparent in large contaminated landscapes where treatment costs are prohibitively expensive in a practical sense (Soares et al., 2021).

Another limitation is that traditional techniques tend to treat contamination as a chemical problem, ignoring the ecological interactions that influence the fate of the pollutant. In the natural environment, contaminants are not static. They are subject to adsorption, leaching, microbial transformation and biological uptake. Physical removal or chemical neutralization does not necessarily restore ecosystem function, since the underlying biological networks are still disturbed (Allouzi et al., 2021). Conventional interventions rarely rejuvenate soil health, microbial diversity, root architecture and nutrient turnover. Consequently, remediated sites may continue to have low productivity, reduced biodiversity, and vulnerability to recontamination. A large challenge in environmental management is the disconnect between pollution removal and ecological system restoration (Zhang et al., 2024).

Limitations of conventional remediation have led to interest in biologically mediated approaches that are compatible with natural ecosystem processes. Bioremediation and phytoremediation have emerged as alternatives because they use living systems to transform contaminants *in situ*. However, plants often have a limited detoxification capacity in the presence of high concentrations of pollutants. Recent findings indicate that most of the observed remediation efficiency of plants is in fact dependent on the associated microbial communities. This realization has shifted research toward plant microbiomes as key players in pollutant degradation and stress adaptation (Ali et al., 2022).

**Figure 1:**
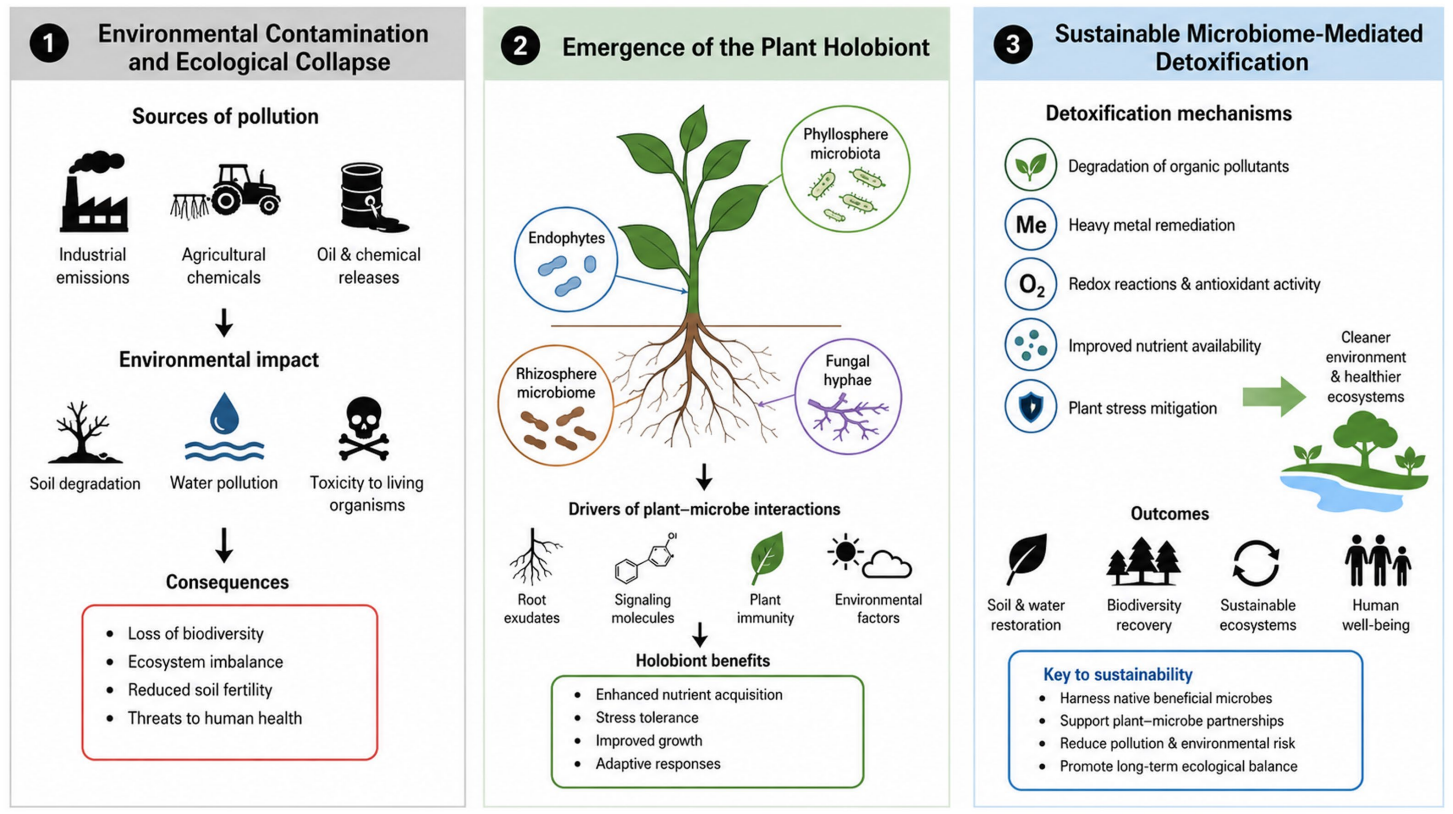
Conceptual transition from pollution-driven ecological degradation to plant holobiont-mediated environmental detoxification. The figure illustrates the limitations of conventional remediation approaches and highlights the emergence of the plant holobiont concept as an integrated ecological detoxification system. Rhizospheric, endophytic, and phyllospheric microbiomes collaboratively regulate pollutant degradation, heavy metal transformation, stress mitigation, and ecosystem recovery through interconnected biochemical and ecological interactions.

The reason for focusing on plant-associated microbiota is based on the necessity to overcome the ecological failures of traditional remediation. The goal is to discover systems that remove contaminants but regenerate functionality of ecosystems. Therefore, understanding why traditional approaches fail provides the necessary foundation for evaluating plant microbiome-based detoxification as an ecologically integrated and scalable solution. To provide a conceptual overview of the progression from environmental contamination toward microbiome-assisted ecological detoxification, a holistic framework illustrating the transition from conventional remediation approaches to plant holobiont-mediated sustainable detoxification is presented in Figure 1 and Table 1.

**Table 1:** Environmental contaminants, ecological impacts, and limitations of conventional remediation strategies.

Environmental contaminant	Major sources	Ecological and biological impacts	Limitations of conventional remediation	Relevance to plant microbiome-mediated detoxification	References
Microplastics and nanoplastics	Industrial discharge, packaging waste, wastewater, agricultural plastics	Soil structural disruption, oxidative stress, microbial dysbiosis, food chain transfer, human toxicity	Physical removal is incomplete; thermal treatment generates secondary pollutants; high operational cost	Rhizospheric and endophytic microbes can adsorb, transform, and biodegrade plastic-associated contaminants while restoring soil ecological balance	Allouzi et al. (2021), Jiang et al. (2020), Meng et al. (2025), Rai et al. (2021)
Heavy metals (Cd, Pb, As, Cr, Hg)	Mining, industrial effluents, fertilizers, sewage sludge	Bioaccumulation, oxidative stress, inhibition of plant growth, disruption of microbial diversity	Excavation and chemical immobilization alter soil chemistry and reduce soil fertility	Plant-associated microbiomes mediate biosorption, chelation, redox transformation, and metal immobilization enhancing phytoremediation efficiency	Rout et al. (2024), Hussain et al. (2025), Liu et al. (2023)
Petroleum hydrocarbons and PAHs	Oil spills, petrochemical industries, vehicular emissions	Toxicity to soil biota, persistence in sediments, mutagenic and carcinogenic effects	Chemical oxidation may generate toxic intermediates; inefficient restoration of ecosystem function	Rhizosphere microbes possessing oxygenases and peroxidases accelerate hydrocarbon biodegradation	Baquero et al. (2022), Tanveer et al. (2024)
Pesticides and herbicides	Intensive agriculture, agrochemical overuse	Toxicity to non-target organisms, alteration of microbial metabolism, groundwater contamination	Soil washing and oxidation are expensive and reduce microbial biodiversity	Microbial consortia degrade organophosphates and xenobiotics through enzymatic biotransformation pathways	Baquero et al. (2022), Tanveer et al. (2024)
Pharmaceutical and personal care pollutants	Hospital discharge, wastewater treatment plants, domestic sewage	Endocrine disruption, antimicrobial resistance, ecological toxicity	Conventional wastewater treatment incompletely removes emerging contaminants	Phyllosphere and rhizosphere microbiomes metabolize pharmaceutical residues and reduce phytotoxicity	Ai et al. (2026), Baquero et al. (2022)
Plastic-associated toxic additives	Synthetic polymers, industrial additives, food packaging	Respiratory toxicity, intestinal inflammation, metabolic disruption	Physical separation does not eliminate associated adsorbed contaminants	Microbial biofilms and rhizospheric interactions facilitate sequestration and detoxification of adsorbed pollutants	Agarwal et al. (2024), Symeonides et al. (2024), Vasse and Melgert (2024)

### 1.2 Emergence of plant microbiomes in detoxification research

One of the most important advances in ecological biotechnology today is the realization that plant microbiomes are active contributors to environmental detoxification. In the past, plants have been studied as single organisms that can absorb, accumulate or transform contaminants through physiological processes (Rueangmongkolrat et al., 2024). Phytoremediation strategies were mainly based on plant characteristics like biomass production, root depth and metal uptake efficiency. But the plant-centric view did not fully explain why some species can thrive in heavily polluted habitats and others cannot, even with similar physiological capacities. Developments in microbial ecology and high-throughput sequencing have revealed that plants harbor highly specific microbial consortia in roots, shoots, leaves and internal tissues, which has fundamentally changed our view of detoxification (Yuan et al., 2023).

Plant microbiomes are complex communities of bacteria, archaea, fungi and other microorganisms that colonize the rhizosphere, phyllosphere and endosphere. These microbes are not passive residents but are actively involved in plant nutrition, stress tolerance, pathogen defense and environmental adaptation. Metabolic capabilities of plant-associated microbes under pollutant stress complement plant detoxification pathways (Sindhu et al., 2022). Many have genes for hydrocarbon degradation, pesticide degradation, metal chelation, and oxidative stress resistance. Many plants possess the ability to metabolize toxic compounds, which often determines their establishment and survival in contaminated environments. Therefore, the fitness of plants under stress is increasingly recognized as a matter of host-microbiome cooperation (Baquero et al., 2022).

This conceptual change led to the holobiont framework, which considers plants and their associated microorganisms as an integrated functional unit. In the contaminated systems root exudates selectively recruit beneficial microbial populations with degrading ability xenobiotics or reducing contaminant toxicity. These microbes in turn enhance nutrient acquisition, phytohormone production and antioxidant defense, allowing plants to tolerate adverse conditions (Liu et al., 2023). Such reciprocal interactions build up a biologically augmented remediation network that is self-sustaining and adaptive. Thus, the plant microbiome is a catalyst of detoxification and a regulator of the ecological resilience in disturbed habitats. Technological advances have greatly benefited the field. With metagenomics, transcriptomics, metabolomics and synthetic microbial ecology, researchers can now identify functional genes, microbial pathways and interaction networks involved in pollutant degradation. These tools have revealed previously undescribed microbial taxa and metabolic capacities associated with detoxification. Such discoveries have transformed the field of remediation science from the use of single strains to the engineering of communities and manipulation of microbiomes (Kline and Joshi, 2024). The reason to study plant microbiomes for detoxification research is that they can link environmental clean-up and ecosystem recovery. The aim is to understand how microbial functions are integrated with plant physiology to generate robust detoxification systems. Hence, this review evaluates ecological interactions, functional mechanisms, and translational opportunities for plant microbiomes and lays the foundation for next-generation sustainable environmental remediation strategies.

## 2 Plant microbiome compartments and ecological niches

Plants are associated with very diverse microbial consortia colonizing different anatomical compartments and ecological interfaces, collectively referred to as the plant microbiome (Tan et al., 2024). These microbial communities include bacteria, fungi, archaea, viruses, and protists that live in the rhizosphere, endosphere, phyllosphere, spermosphere, and other specific niches where they interact dynamically with host plants and the surrounding environment. The structural organization of these microbiomes is not random, but rather reflects complex ecological filtering processes governed by plant genotype and developmental stage, physicochemical properties of the habitat, nutrient availability, climatic conditions and anthropogenic disturbances (Coolen et al., 2024). Over the past few years, a growing interest has been focused on understanding the role of compartment-specific microbial communities related to the processes of environmental detoxification, such as biodegradation of organic pollutants, immobilization of heavy metals, transformation of nutrients, and restoration of ecological balance in contaminated ecosystems (Li Q. et al., 2025). Because different plant compartments offer unique physicochemical conditions, they harbor microbial populations with distinct taxonomic compositions and functional capacities, thereby creating specialized ecological niches that collectively enhance plant resilience and environmental sustainability (Tanveer et al., 2024).

### 2.1 Rhizosphere microbial communities

The rhizosphere is one of the most metabolically diverse and biologically active microbial habitats of all plant-associated habitats found in terrestrial ecosystems (Hussain et al., 2025). The rhizosphere is the narrow zone of soil affected by root exudates and root-associated biological activity and is characterized by dense microbial populations often several orders of magnitude higher than in bulk soil. Root exudation is the main driver of rhizospheric microbial assembly by the release of a variety of sugars, amino acids, organic acids, phenolics, flavonoids, vitamins and secondary metabolites into the surrounding soil matrix. Consequently, the rhizosphere serves as a highly selective ecological niche where cooperative and competitive microbial interactions continuously influence microbial diversity, community stability, and functional efficiency (ul Malook et al., 2025).

Many rhizobacteria and mycorrhizal fungi are remarkably capable of degrading xenobiotic compounds such as polycyclic aromatic hydrocarbons, pesticides, herbicides, petroleum hydrocarbons, industrial dyes and pharmaceutical residues and produce extracellular enzymes such as laccases, peroxidases, oxygenases, dehalogenases and hydrolases which catalyze the transformation of toxic contaminants to less harmful intermediates (Nizamani et al., 2025). Furthermore, several rhizospheric bacteria exhibit metal-resistant traits that enable them to tolerate and transform heavy metals through biosorption, bioaccumulation, precipitation, redox transformation, and chelation mechanisms. Genera such as *Pseudomonas*, *Bacillus*, *Rhizobium*, *Burkholderia*, and *Azospirillum* have been extensively reported for their ability to improve phytoremediation efficiency in heavy metal-contaminated soils (Hu et al., 2022).

The different plant growth-promoting rhizobacteria produce phytohormones like indole-3-acetic acid, gibberellins and cytokinins, while others assist in nutrient acquisition through nitrogen fixation, phosphate solubilization and siderophore production. The adverse effects can be ameliorated by rhizospheric microorganisms through the enhancement of antioxidant defense systems, reduction of ethylene-induced stress through ACC deaminase activity and improvement of root architecture for better nutrient and water uptake (Ai et al., 2026).

Microbial communities under pollutant stress are selectively enriched in genes involved in stress tolerance, efflux pumps, hydrocarbon degradation and metal resistance pathways. Such adaptive responses indicate that the rhizosphere is an evolutionary reservoir of catabolic potential that is capable of responding rapidly to environmental perturbations (Bradley et al., 2022). Interestingly, the functional redundancy found in rhizospheric microbiomes is one of the factors that contribute to ecosystem resilience, allowing the continuity of key biochemical processes, even in changing environmental conditions. These findings highlight the ecological importance of rhizosphere microbiomes as key mediators of plant-facilitated environmental remediation (Greeshma et al., 2022).

### 2.2 Endophytic and phyllospheric microbiomes

In addition to the rhizosphere, plants are colonized by complex microbial communities on the internal tissues and aerial surfaces, resulting in endophytic and phyllospheric microbiomes, which are highly specialized ecological niches (Jing et al., 2025). Despite the environmental constrains, phyllospheric microbial populations constitute one of the largest microbial reservoirs on Earth and they play an important role in plant adaptation and ecosystem functioning (Saati-Santamaría et al., 2023).

Many of the endophytic fungi and bacteria known to be present in plants are able to metabolize toxic organic compounds absorbed by the plant through the soil as well as through water in the case of aquatically grown plants that have been contaminated. For example, endophytic fungi and bacteria are known to be able to degrade benzene, toluene, ethylbenzene, xylene, and chlorinated hydrocarbon compounds and have been isolated from several plant species growing in contaminated soils and/or aquatics (Xiong et al., 2021a). By degrading toxic compounds prior to their reaching concentrations that could be detrimental to the host plant, endophytes are believed to lower phytotoxicity. Likewise, endophytes have been reported to produce antioxidant enzymes, osmotic agents, and secondary metabolites that enable the host plant to respond to oxidative stress resulting from exposure to pollutants in the environment (Roy et al., 2025).

Endophytes have ecological significance when used in phytoremediation systems, where the efficiency of removing contaminants from soil through plant uptake is significantly increased with endophytic microorganisms. Additionally, several endophytic microorganisms can regulate the expression of detoxification enzymes, metal transporters, and stress response proteins in plants, providing plants and endophytes with new opportunities to adapt to polluted environments. Furthermore, many fungal endophytic microorganisms such as Trichoderma, Penicillium, and Piriformospora have shown great potential for improving plant tolerance to salinity, drought, and heavy metals, which demonstrates the multifunctionality of endophyte communities in enhancing ecosystem resilience (Liu et al., 2021).

In addition to the contribution of endophytic microorganisms to ecosystem resilience, the phyllosphere represents a unique microbial community affected by the adverse abiotic conditions of the environment, as well as its close association with airborne pollutants and biologically sourced particulate matter. As the primary interface between atmospheric and terrestrial ecosystems, the surface of the leaves provides an important surface area for microorganisms in the phyllosphere to degrade airborne pollutants, including volatile organic compounds, methane derivatives, and hydrocarbons released into the atmosphere by plant metabolism or through human activities (Moroenyane et al., 2021). Certain microorganisms that inhabit the phyllosphere have the metabolic capacity to utilize methanol, formaldehyde, and other volatile compounds produced by plants or as a result of human activities. These metabolic functions have implications for atmospheric detoxification and broader biogeochemical cycles (Wang et al., 2023).

Microorganisms within the phyllosphere create biofilms, produce pigments that protect against ultraviolet light, create antimicrobial proteins, and alter how plants regulate their stomata. All of these actions contribute to maintaining the integrity of leaf surfaces in this ecosystem, in addition to influencing the exclusion of pathogens and priming the immune system of a plant to combat disease that arises from being exposed to environmental stressors (e.g., heat, sunlight, etc.). The composition of the phyllosphere is highly dynamic, and responds to variable climatic conditions such as humidity, temperature, rainfall, and air quality. The phyllosphere is, therefore, sensitive to change in the environment (Grzyb and Szulc, 2024).

**Figure 2:**
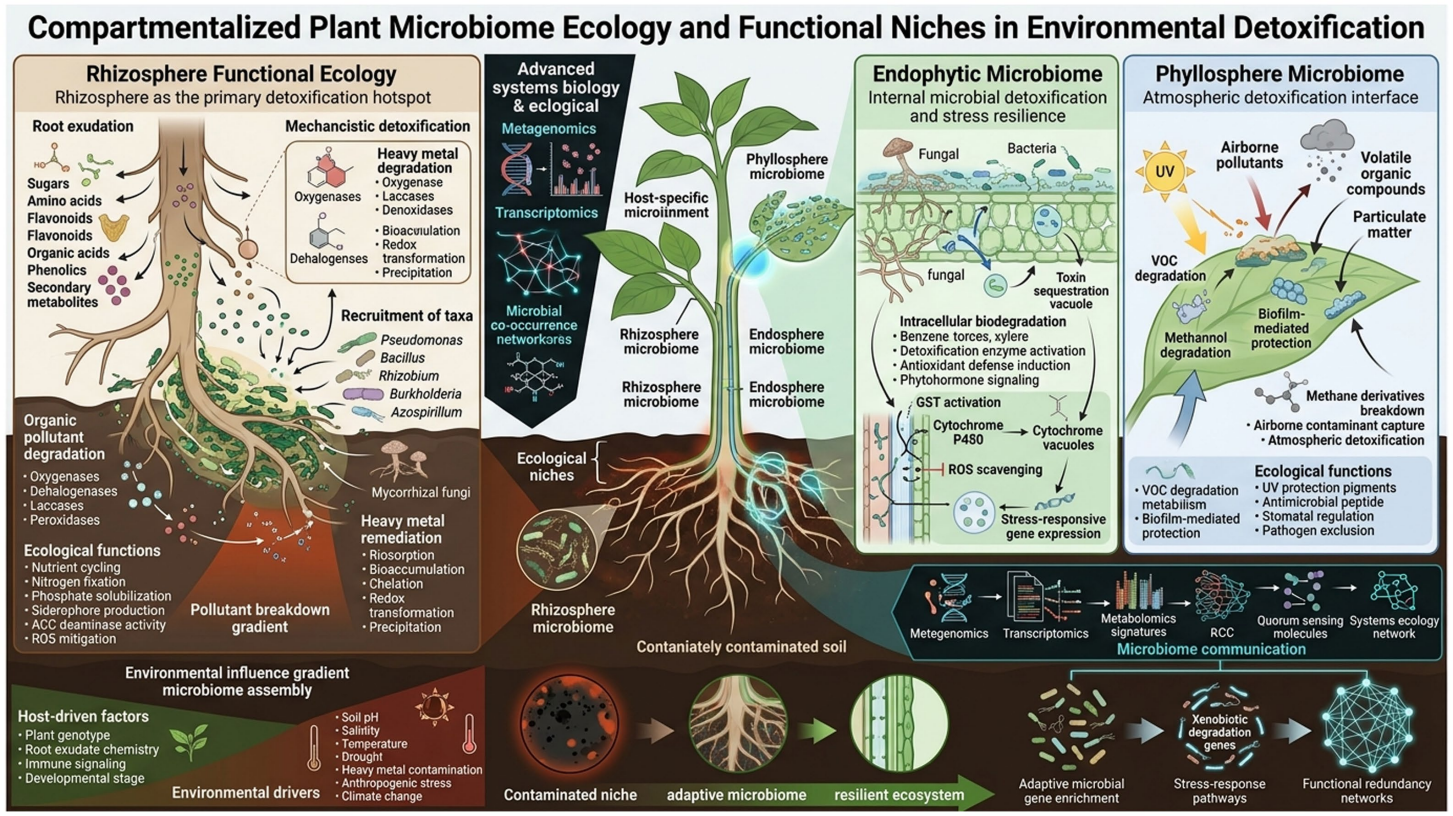
Compartment-specific organization and ecological-functional roles of plant microbiomes in environmental detoxification. The figure depicts the spatial distribution of rhizospheric, endophytic, and phyllospheric microbial communities and their coordinated contributions to xenobiotic degradation, heavy metal sequestration, nutrient cycling, stress tolerance, and ecological resilience. Host-driven recruitment and environmental filtering collectively shape microbiome assembly and functionality.

Thus, plant microbiomes must be considered together as one combined ecological system; rather than three separate compartments, and collectively contribute to plant health and environmental detoxification (Otlewska et al., 2020). Considering the compartment-specific organization of plant-associated microbial communities, the ecological and functional distribution of rhizospheric, endophytic, and phyllospheric microbiomes involved in environmental detoxification is summarized in Figure 2 and Table 2.

**Table 2:** Ecological niches of plant microbiomes and their functional roles in environmental detoxification.

Plant microbiome compartment	Dominant microbial groups	Ecological characteristics	Major detoxification functions	Representative functional mechanisms	References
Rhizosphere	Pseudomonas, Bacillus, Rhizobium, Burkholderia, AM fungi	Nutrient-rich root-influenced soil zone with intense microbial interactions	Degradation of hydrocarbons, pesticide detoxification, heavy metal immobilization	Oxygenase production, ACC deaminase activity, siderophore secretion, biosorption, quorum signaling	Sindhu et al. (2022), Xiong et al. (2021a), Zhuang et al. (2020)
Endosphere	Endophytic bacteria and fungi (Trichoderma, Penicillium, Piriformospora)	Internal plant tissues with stable host-associated interactions	Intracellular biodegradation of xenobiotics and stress mitigation	Antioxidant enzyme induction, phytohormone regulation, detoxification gene activation	Gouda et al. (2018), Huang et al. (2026), Shaffique et al. (2022)
Phyllosphere	Methylotrophs, epiphytic bacteria, yeasts, filamentous fungi	UV-exposed aerial leaf surfaces with fluctuating humidity and nutrient limitation	Atmospheric pollutant degradation and volatile organic compound metabolism	Biofilm formation, VOC metabolism, methanol utilization, UV-protective metabolite production	Ai et al. (2026), Hu et al. (2022)
Spermosphere	Seed-associated bacteria and fungi	Early developmental niche influencing seed germination and microbiome inheritance	Early-stage stress resistance and microbial recruitment	Vertical microbial transmission, antimicrobial metabolite secretion	Moroenyane et al. (2021), Saati-Santamaría et al. (2023)
Root-Endosphere Interface	Symbiotic fungi and diazotrophic bacteria	Highly selective host-controlled niche	Enhanced nutrient acquisition and detoxification synergy	Nitrogen fixation, phosphate solubilization, metal sequestration	Li F. et al. (2022), Yue et al. (2024)
Holobiont-Level Microbiome Network	Cross-compartment microbial consortia	Integrated ecological network regulated by host genotype and environment	Functional resilience under environmental stress and restoration of ecosystem stability	Functional redundancy, microbial networking, adaptive metabolic plasticity	Grzyb and Szulc (2024), Roy et al. (2025), Xing et al. (2023)

### 2.3 Host specificity and environmental drivers

Microbial communities in plants are influenced by the plant itself (host-specificity) and the environment; the host-environment interaction will affect how and where the microbe is attracted to, survives, and performs in the plant ecosystem. When a plant has characteristics that favor attracting specific microbe groups, we call this host-specificity due to the fact that only specific plants can attract and be associated with the same type of microbe (Huang et al., 2026). Within the plant, the interaction of host characteristics (genetic background, immune responses, root exudate chemistry, etc.) with microbe characteristics (genomes, stress response/behavioral attributes to individual plant hosts, etc.) contributes to the unique and co-evolutionary association between them; therefore, plants with the same specific genotype may or may not provide an environment that will result in the same specific microbe population due to their unique characteristics that fluctuate over long periods of time due to variations in environmental factors (e.g., temperature, moisture, etc.) (Xiong et al., 2021b).

Genetic differences between different genotypes of the same plant species influence microbial community assemblages by altering the abundance and diversity of root exudation (e.g., growth, nutrient composition, etc.) and the levels of plant secondary metabolites (i.e., phenolics, terpenoids, flavonoids) and other compounds (defense-related, etc.), thus modifying the microbial diversity and type of ecological interactions within a plant (Li F. et al., 2022).

Soil chemistry, or physicochemical characteristics such as soil pH, organic matter content, nutrient availability, moisture level, salinity, and redox potential have important effects on microbial activity and survival (Van Nuland et al., 2023). Among these soil characteristics, soil pH is a well-studied driver of microbial richness and is known to influence microbial diversity by affecting microbial competition, enzyme activity, and solubility of nutrient species. Additionally, environmental factors such as temperature, precipitation levels, ultraviolet light, and seasonal weather patterns will generally modify microbiota richness indirectly by modifying microbial growth rate(s) and/or ecological interactions. Because of the above factors, available evidence suggests that different ecosystems are likely to support grossly different distributions of microbes associated with the roots of plants (Yue et al., 2024).

In addition to the previously-mentioned factors, anthropogenic disruption is one of the most important environmental drivers of microbiome composition. Anthropogenic disturbance will often disrupt the composition of the native microbial community, thereby reducing the stability of the affected ecosystem (Xing et al., 2023). In contrast, anthropogenic disturbance may also encourage enriched populations of stress-tolerant microorganisms capable of degrading a variety of toxins through specialized detoxification pathways. In addition, evidence of increased abundance of genes related to xenobiotic degradation, oxidative stress tolerance, membrane transport, and metal sequestration among microbial populations that inhabit a contaminated habitat indicate that ecological acclimation to these types of perturbations/recolonization represent the resilient and flexible adaptation of plant-associated microbiomes over time (Zhuang et al., 2020).

In heavily contaminated environments, species of plants that can recruit microbial consortia that have adapted to survive under stressful conditions exhibit superior capacity for remediation and enhanced plant physiological tolerances than species that do not have the ability to recruit such consortia of microbes (Dong et al., 2022). On the other hand, if plants are exposed to an environmental disturbance that disrupts or destabilizes their association with these beneficial microbes, their ability to remain healthy will be compromised as well as the ability of the plants to remediate their environment. Therefore, having an understanding of these intricate interactions is critical to developing microbiome-based strategies that will lead us toward restoring our environment in a sustainable manner (Yuan et al., 2022).

With recent advances in systems biology, ecological modeling and the synthetic engineering of microbiomes, research is being conducted into methods that can be used to manipulate plant-associated communities of microbes in order to enhance the resilience of ecosystems and their capacity for detoxification (Strand, 2022). Using knowledge of plant-microbe specificity in conjunction with environmental ecology, it is becoming more common for researchers to design targeted consortia of microbes that enhance the ability of plants to perform phytoremediation, carbon sequestration, provide nutrient cycling and adapt to environmental stress. Ultimately, the study of the plant microbiome compartments and ecological niches will provide critical information about the complex biological networks that govern the functionality of ecosystems and their health (Naing et al., 2021).

## 3 Functional mechanisms in environmental detoxification

The plant microbiome is made up of bad and neutral tiny living things. The good ones, like plant growth-promoting bacteria help plants grow. Plant growth-promoting bacteria do this in two ways. Some plant growth-promoting bacteria make helpers, like auxins, cytokinins and gibberellins (Timofeeva et al., 2024; Swain et al., 2025). These helpers affect how plants grow by changing the amount of hormones that the plant itself makes when it is working with plant growth-promoting bacteria.

In addition to this some types of plant growth-promoting bacteria (PGPB) release an enzyme called 1-aminocyclopropane-1-carboxylate (ACC) deaminase (Khatoon et al., 2024, Tariq and Farhat, 2025). This enzyme decreases the stress hormone ethylene in plants. Several bacteria, like *Pseudomonas, Arthrobacter* and *Bacillus* make plants grow better (Shahid et al., 2023; Albureikan, 2023). They do this by producing ACC deaminase. Bacteria like *Pseudomonas*, *Paraburkholderia* and *Pantoea* have been found on wheat and soybean roots (Solanki et al., 2025; Zhang et al., 2023). These bacteria help plants grow by doing things like making phosphate easy for plants to use fixing nitrogen making acetic acid and producing ACC deaminase. PGPB like Pseudomonas, *Arthrobacte*r and *Bacillus* are known to make plants grow better. They produce ACC deaminase which helps reduce stress in plants caused by ethylene. The bacteria *Pseudomonas*, *Paraburkholderia* and *Pantoea* play a role, in promoting plant growth (Solanki et al., 2025; Zhang et al., 2023; Biswas et al., 2024). They do this by producing ACC deaminase and helping plants get the nutrients they need.

### 3.1 Microbial degradation of organic pollutants

The microorganisms living in soils of varying textures and depths in the forest ecosystem contribute significantly to the breakdown of organic pollutants by serving as natural decomposers (Compant et al., 2019; Li H. et al., 2025). The enzymes produced by them form the basis of this breakdown because enzymes are biological catalysts that break down organic pollutants to simpler forms (Kour et al., 2021; Thijs et al., 2016). This is achieved through the enzymes breaking specific bonds or functional groups in the molecules of the organic pollutants. The effectiveness of this process depends upon the nature of the pollutants, which may be petroleum hydrocarbons, and even man-made substances such as pesticides and drugs. Each enzyme works on a different type of bond or functional group in the molecules of pollutants (Saravanan et al., 2021).

**Figure 3:**
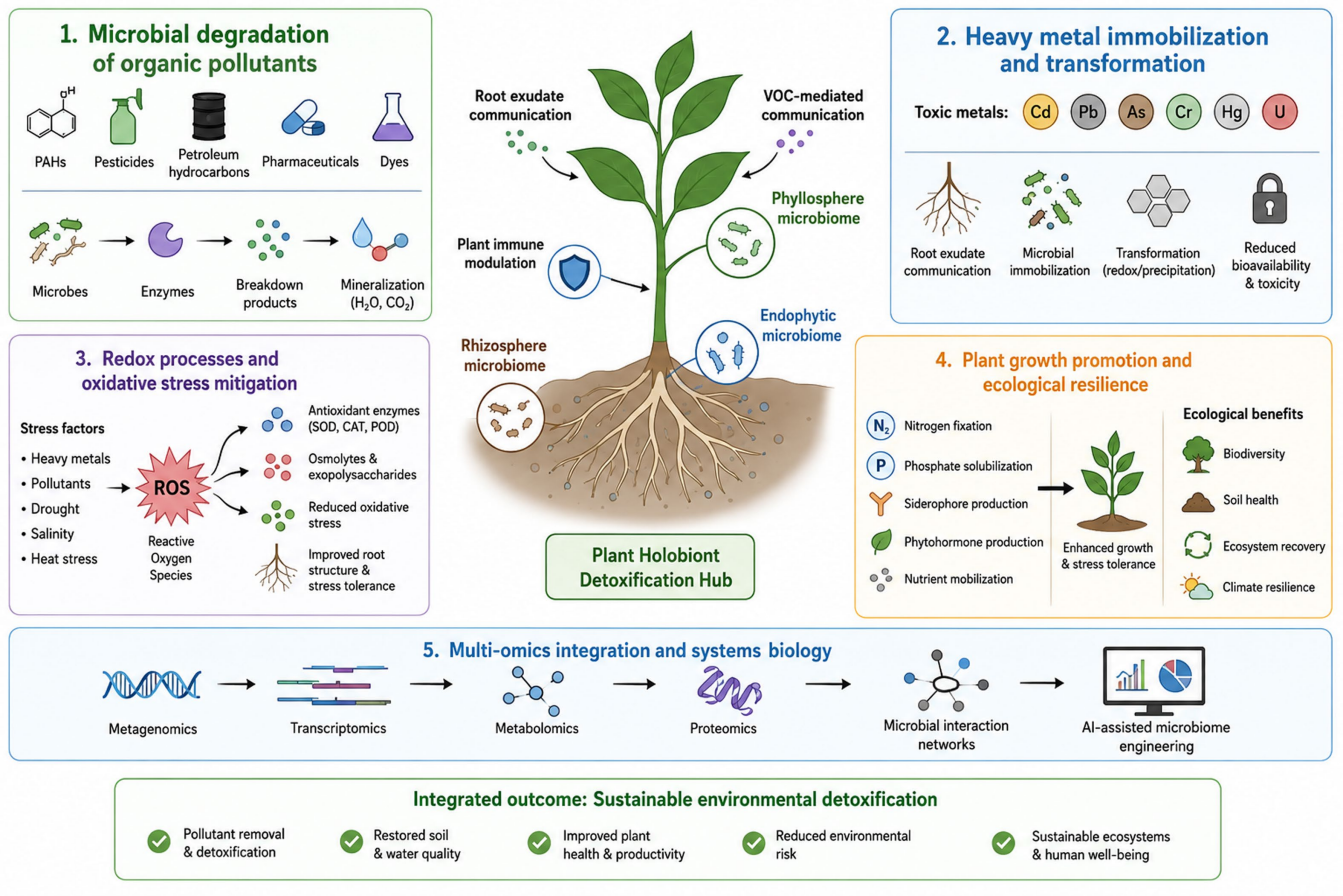
Integrated functional mechanisms of plant microbiomes in environmental detoxification. The figure illustrates interconnected microbiome-mediated detoxification pathways including xenobiotic biodegradation, heavy metal immobilization, oxidative stress mitigation, nutrient mobilization, and plant growth promotion. Rhizospheric, endophytic, and phyllospheric microorganisms collectively regulate ecological resilience through coordinated biochemical, metabolic, and signaling interactions.

Some types of microbes have evolved unique systems of co-metabolism for the decomposition of certain hazardous materials such as polycyclic aromatic hydrocarbons (PAHs) and chlorinated compounds (Lin et al., 2022). Moreover, the use of microbial communities based on synergistic activity among different types of microbes can result in improved efficiencies of microbial decomposition processes as shown in Figure 3. Soil pollutants’ decomposition by microorganisms is an extremely complicated process requiring multiple kinds of organisms and enzymes. Thus, microbial degradation of PAHs is primarily done by such fungi as *Fusarium oxysporum* (Ascomycetes) and *Laccaria bicolor* (Basidiomycetes) as well as such bacteria as *Streptomyces griseus* (Actinobacteria). Microbes use oxygenase enzymes to break down PAHs’ aromatic rings making them decomposable (Liu P. et al., 2025).

The tiny things that microorganisms make, called enzymes are really important for breaking down things in the soil. There are kinds of enzymes like oxygenases that help get rid of certain types of bad things like the kind found in gasoline and other chemicals. Oxygenases are really good at breaking down these things like the kind that have lots of rings called PAHs. Other enzymes, like dehydrogenases are also very important. They help make the bad things less bad by changing them (Lü et al., 2024). This helps the microorganisms break down the things like the kind found in cleaning products and medicines. The enzymes do this by helping things get bigger or smaller which makes it easier for the microorganisms to break them down. There are also enzymes called peroxidases, made by things like fungi that’re really good at breaking down bad things like pesticides. The fungi, like *Phanerochaete chrysosporium* make these enzymes to help break down the things into things that are not bad, for us. This is really helpful because it makes the bad things less toxic (Lü et al., 2024). The integrated biochemical and ecological mechanisms through which plant-associated microbiomes mediate pollutant detoxification and stress resilience are mechanistically illustrated in Figure 3.

### 3.2 Roles of laccases, peroxidases, and oxygenases in the rhizosphere during the breakdown of organic pollutants

Laccase is a blue-colored multicopper enzyme which functions in the polymerization process by forming dimers, degradation of polymers, and breakdown of aromatic rings. This enzyme is widely found in higher plants and fungi; specifically in Ascomycetes, Deuteromycetes, and Basidiomycetes groups, and it is found abundantly in the white-rot fungi responsible for the degradation of lignin. Laccase enzymes are used in the synthesis of many organic compounds where the common substrates are phenols and amines. By oxidation reaction, the intermediates produced are dimers and oligomers. Laccases have become extremely important in recent times, owing to their many uses in different industries including textiles, paper, and foods. They are also used extensively in developing biosensors, biofuel cells, medical diagnostic devices, and even in the biodegradation of herbicides, pesticides, and explosives from the soil. Due to the ability of laccases to oxidize not only phenolic but also non-phenolic lignin-like products as well as environmental contaminants, they have gained much attention over the last few decades (Li, et al., 2021).

The peroxidases represent a multifunctional enzyme that has wide applications in environmental and industrial biotechnology. These enzymes participate actively in the detoxification of water polluted with phenols, cresols, and chlorinated phenols. Peroxidases are involved in biopulping and biobleaching in the paper manufacturing industry. These enzymes find applications in degrading textile dyes and in the decomposition of remaining peroxide in food materials. The effluent generated by the textile industries contains a number of dyes that resist conventional bleaching and can be easily oxidized by peroxidase enzymes. The high capacity of the white-rot fungi for the breakdown of lignin is due to the non-specific free-radical oxidation reactions performed by the peroxidase enzymes released by these organisms (Chandra et al., 2011). These enzymes show oxidative capabilities on a wide variety of substrates, including dimethoxy benzene, lignin dimers, phenols, amines, dyes, aromatic alcohols, and both phenolic and non-phenolic substrates. Moreover, dye decolorizing peroxidase enzymes produced by Agaricus-type fungi are able to oxidize a range of dyes and phenolic substances. Because of their fairly unspecific enzyme action, the peroxidases allow white-rot fungi to break down various types of environmental contaminants, such as dioxins, polychlorinated biphenyls (PCBs), petroleum hydrocarbons, explosives like TNT, industrial dye wastes, herbicides, and pesticides (Basumatary et al., 2023).

This microbiome has been referred to as the “second genome” of plants due to its key importance in increasing plant growth, development, and fitness. In defense against pathogens, plants deploy a wide range of immune processes, which inhibit pathogen infection and change the profile of the plant root exudates. Changes in root exudates include changes in polysaccharides, proteins, amino acids, organic acids, phytohormones, and phenols, thus promoting the recruitment of beneficial microbes in the rhizosphere (Trivedi et al., 2020; Afridi et al., 2024). Beneficial microbes help protect the host plants through a variety of processes. These processes include competition for habitats and resources, regulating plant development, and stimulating immunity (Liu Y. et al., 2025). Recent studies have indicated significant differences in the profile of microbial communities inhabiting the rhizospheres of plants carrying mutations in the rbohD gene compared to wild-type plants. This observation suggests that ROS may affect the microbial community structure in plant roots (Pfeilmeier et al., 2021). However, it is uncertain whether the effect of ROS on rhizospheric microbiomes is direct or occurs indirectly via ROS-mediated changes in plant development and exudation.

### 3.3 Microbial mediation of heavy metal immobilization and transformation

The contamination of soil by heavy metals (HMs) has become one of the environmental issues globally because of persistence, toxicity, and resistance. The toxic HMs may disturb ecological balance and cause negative effects on living organisms. Besides, these metals could also find ways into human bodies via food contaminated with HMs. Therefore, it is critical to clean up soils contaminated by HMs. One of the effective solutions for addressing the negative effect of HMs is through the application of microorganisms. In the past, microorganisms played a crucial role in the process of recovering degraded ecosystems, which had persistent effects on the environment. The use of bioremediation techniques decreases mobility of HMs and assists in extracting heavy metals from the environment. Therefore, it is now possible to apply bioremediation to clean up HM-contaminated soils owing to scientific breakthroughs. In this regard, there are several approaches used by microorganisms to minimize the negative effect of HMs (Tang et al., 2024).

Bioremediation is a method that helps remove HMs. ions from areas. It uses biological techniques like adsorption, bioaccumulation, biotransformation and bioleaching to get rid of toxic metal ions. These techniques use materials such as bark, coconut shell, rice shell, wheat shell, seaweed, seeds, aquatic plants, agricultural waste, microorganisms. Microorganisms play a role in bioremediation (Sharma et al., 2022). They can change the form of metals, which affects how easily they dissolve become available and move in soil and water. HMs. can be made less mobile by microorganisms through complex processes like oxidation–reduction reactions chelation changes in metal complexes biomethylation. Bioremediation and microorganisms are very effective, in eliminating heavy metal ions. Microorganisms help in making heavy metals less toxic. The process of bioremediation uses microorganisms to remove metals (Pande et al., 2022).

It is important to highlight that microbial enzymatic catalysis plays a critical role in transforming metals from higher oxidation states to lower oxidation states. For example, the way *Thiobacillus ferrooxidans* and *T. thiooxidans* break down uranium is an example of this. We can find microorganisms that break down metals in both places with air and without air but aerobic microbes are better at cleaning up the environment than anaerobic microorganisms. Microorganisms use ways to move heavy metals around and change them into forms that are not harmful which is really important for them to survive in places with a lot of metal pollution (Gunjyal et al., 2023). Microorganisms like metals use several methods to deal with the bad effects of heavy metals and make sure they can keep living. These methods include biosorption, bioaccumulation, biotransformation and bioleaching which help microorganisms survive in a metal-polluted environment. It is very important to note that using these methods has been shown to be effective in cleaning up the environment, which shows how good microorganisms are at reducing the bad effects of heavy metals, on the environment (Agarwal et al., 2024).

### 3.4 Biotic and abiotic stress mitigation

Phyto-microbes, as referred in the term, are a vast variety of bacteria, archaebacteria, fungi, and viruses that have an extremely important ecological association with the host plant, thus making up the phyto-microbiome (Table 3). Not only does the phyto-microbiome facilitate the growth and development of the host plant in normal conditions but also ensures that it maintains its homoeostasis during both biotic and abiotic stress conditions. As a result of its highly efficient metabolic nature, phyto-microbiome aids the host plant in adapting to abiotic stresses by synthesizing antioxidants, plant growth hormones, and active molecules along with helping the host plant detoxify itself from any potentially harmful substance from its environment and scavenge for any reactive oxygen species or any free radicals. In the context of abiotic stress management, phyto-microbes improve plant tolerance to drought, salinity, extreme temperatures, heavy metal toxicity, nutrient deficiency, and flooding through mechanisms such as enhanced nutrient acquisition, modulation of stress-responsive signaling pathways, production of exopolysaccharides, regulation of ethylene levels via ACC deaminase activity, and induction of antioxidant defense systems. These microbial-mediated responses contribute to improved water-use efficiency, maintenance of cellular osmotic balance, protection of photosynthetic machinery, and stabilization of plant metabolism under adverse environmental conditions. Beyond abiotic stress mitigation, the phyto-microbiome also serves as a critical component of biotic stress management by protecting plants against pathogenic bacteria, fungi, viruses, nematodes, and insect pests. Beneficial microbes suppress pathogen colonization through competition for nutrients and ecological niches, production of antimicrobial metabolites, siderophores, lytic enzymes, and volatile organic compounds (VOCs), as well as by activating induced systemic resistance (ISR) and systemic acquired resistance (SAR) pathways within the host plant. These defense mechanisms strengthen plant immunity and reduce disease incidence while promoting sustainable crop productivity. With extensive information regarding the structure and functioning of the phyto-microbiome along with the exact mechanism through which abiotic stresses are relieved with the help of the phyto-microbiome, its use in alleviating abiotic stress among crops will be achieved (Anzano et al., 2022; Arun et al., 2020).

**Table 3:** Redox processes and exact mechanisms of plant microbiome-mediated biotic and abiotic stress mitigation.

Abiotic stress	Redox process in plant microbiome	Exact mechanism of action	Stress mitigation outcome	Microbial examples	References
Drought stress	ROS detoxification through antioxidant enzymes	Microbes produce superoxide dismutase (SOD), catalase (CAT), and peroxidases that convert superoxide radicals (O₂^−^) and H₂O₂ into water and oxygen	Prevents lipid peroxidation and protects chloroplast membranes	*Bacillus*, *Pseudomonas*, *Azospirillum*	Zhakypbek et al. (2026)
Salinity stress	Glutathione–ascorbate redox cycle activation	Microbial inoculation increases glutathione reductase and ascorbate peroxidase activities, maintaining cellular redox balance under excess Na^+^	Maintains ion homeostasis and membrane stability	PGPR, arbuscular mycorrhizal fungi	Selvakumar et al. (2013)
Heat stress	Induction of antioxidant metabolites	Endophytes stimulate production of flavonoids, carotenoids, and phenolics that neutralize ROS generated during high temperature	Reduces oxidative protein denaturation and cellular injury	*Trichoderma*, *Piriformospora indica*	Gill et al. (2016)
Cold stress	Redox-mediated osmolyte regulation	Microbial signaling enhances proline and glycine betaine synthesis via redox-sensitive pathways	Protects enzymes and membranes against chilling injury	*Rhizobium*, *Serratia*	Kour and Yadav (2023), Liu et al. (2019)
Heavy metal stress	Reductive transformation of toxic metals	Microorganisms reduce metal ions such as Cr^6+^ to less toxic Cr^3+^ using oxidoreductase enzymes	Decreases metal toxicity and oxidative stress	*Pseudomonas fluorescens*, *Enterobacter*	Subrahmanyam et al. (2018), Wasi et al. (2008)
Oxidative stress	Enhancement of glutathione biosynthesis	Microbes stimulate glutathione accumulation which directly scavenges ROS and repairs oxidized proteins	Restores intracellular redox equilibrium	PGPR and endophytic bacteria	Thanwisai et al. (2022)
Nutrient deficiency stress	Electron transfer and redox cycling in rhizosphere	Rhizospheric microbes facilitate Fe^3+^ reduction to Fe^2+^ and improve nutrient solubilization through redox reactions	Enhances nutrient availability and uptake	*Bacillus subtilis*, *Rhizobium*	Dudeja et al. (1997), Zhou et al. (2018)
Flooding or water logging stress	Regulation of nitric oxide (NO) signaling	Microbial NO production activates anaerobic response genes and antioxidant defense pathways	Improves tolerance to hypoxia and root damage	Denitrifying bacteria, *Azospirillum*	Hamonts et al. (2013), Salazar-Garcia et al. (2022)
UV radiation stress	Redox-induced secondary metabolite production	Microbial symbionts stimulate accumulation of anthocyanins and phenolics that absorb UV and neutralize ROS	Protects DNA and photosynthetic apparatus	Endophytic fungi and cyanobacteria	Giza et al. (2025)
Multiple abiotic stresses	ACC deaminase-mediated ethylene reduction	Microbial ACC deaminase cleaves ACC, reducing stress ethylene synthesis and associated ROS burst	Promotes root growth and stress resilience	*Variovorax*, *Bacillus subtilis*	Abd-Elhalim et al. (2024)
Soil alkalinity/acidity stress	Regulation of cellular redox buffering	Microbes modulate NADH/NAD^+^ and glutathione redox status to stabilize intracellular pH and metabolism	Maintains metabolic activity under pH stress	Rhizosphere bacterial consortia	Cui et al. (2024)
Combined drought and salinity stress	Biofilm-associated redox protection	Microbial biofilms enhance antioxidant accumulation and create a protective rhizospheric microenvironment	Improves water retention and oxidative stress tolerance	Biofilm-forming PGPR and fungi	Fazal et al. (2025), Al-Turki et al. (2023)

## 4 Ecological and functional dynamics of the phyto-microbiome in plant stress adaptation and resilience

Phyto-microbiome is another term that can be used to describe the different types of microbes, comprising bacteria, archaea, fungi, and viruses, which have established a symbiotic, mutualistic, or parasitic relationship with the plant kingdom (Khan, 2023; Patyal et al., 2025; Nadarajah and Abdul Rahman, 2021). There are various types of microorganisms depending on their relationship with plants. The first type is endophytes that are microbes living inside the plant tissue while others are epiphytes, which are microbes living on the surface of plants’ various parts. Rhizoplane and phylloplane provide conducive living conditions for the microorganisms.

However, compared to the other microorganism communities, rhizospheric microbiome heavily depends on host plants through rhizodeposition. The different chemicals released by the host plants include amino acids, carbohydrates, organic acids, fatty acids, siderophores, and flavonoids. These chemicals act as signals that allow interactions between the host plant and the specific microbes. Different plants release various chemicals; therefore, root exudation is plant-specific. Various factors affect root exudation, and they include plant genetics, plant immunity, signaling pathway, and environmental factors. The rhizosphere, or soil surrounding plant roots, is a highly dynamic environment enriched with nutrients, minerals, and other compounds that support diverse bacterial populations. Plant roots release bioactive compounds known as root exudates, which influence the composition and activity of rhizospheric microorganisms. These microbial communities play essential roles in plant health, with beneficial groups such as plant growth-promoting rhizobacteria (PGPR) enhancing plant growth and improving resilience under stress conditions. The plant-growth-promoting rhizobacteria do this by making special helpers called phytohormones. They also lower the levels of things, like ethylene oxide and help the plant deal with not having enough water. The plant-growth-promoting rhizobacteria even help the plant make special cleaners called enzymes (Kong and Six, 2012). The phyllosphere provides an optimal habitat for microorganisms which comprise a wide array of microorganisms which are advantageous to the plant. It includes bacteria, fungi, and viruses. The function of the plant depends on the microorganisms present in the phyllosphere. They help in removing any contaminants from the plant. Furthermore, they aid in maintaining the health of the plant (Arun et al., 2020). There are fungi found in rhizospheric soils which exhibit high degradative abilities when it comes to pollutant reduction, thus ensuring protection for the plants against any abiotic stress (Shourie and Vijayalakshmi, 2022). There are fungi such as the Arbuscular mycorrhizal fungi (AMF), which are obligate mycorrhizal fungi, that engage into symbiosis with the vascular plants including the halophytes. AMFs have the ability to produce sporangia in the rhizospheric environment as well as develop vesicles and hyphae in the roots. Hyphal development by AMFs ensures that plant growth is enhanced through high access to soil surface area. Nutrient transport by AMFs ensures improved nutrition for the plants (Shourie and Vijayalakshmi, 2022). The endophytes live with the host plants. They stay with the host plants for as long as the host plants live. The way it works is that endophytes usually get into seeds, roots, leaves and stems of the host plants. Endophytes are really good for the host plants because they help the host plants grow by doing things like helping the host plants get nitrogen making more phytohormones and taking in more nutrients. When the host plants are under a lot of stress from things that are not living like much salt or water the endophytes help the host plants grow by doing things like collecting osmolytes making the host plants stronger making phytohormones like ABA, GA, cytokinin and IAA and making ACC deaminase to reduce ethylene (Pal et al., 2021).

### 4.1 Community assembly and functional redundancy

It is widely accepted that functionally redundant biological systems may explain the known connection between high diversity of microbial communities colonizing the human body and human health. All of the processes involved in the development of microorganisms in their environments are included in the assembly of microbial communities. Establishment of microbial communities can be influenced by many factors including the host’s genotype, nutrition available, habitat, microbe-microbe interaction and ecological selection. The combination of stochasticity (stochastic colonization and dispersal) and determinism (selective pressures acting from the host and ecosystem) determines the microbiome assembly in both plants and animals. The microbiomes contribute greatly to nutrition recycling, physiological functions of the host, ecological stability and stress tolerance (Methorst et al., 2021). The microbiome includes microbes which can perform the same functions. This can be termed as “redundancy” in the microbiome. If one species of microbe gets weaker, other microbes that can do similar things take its place. This will help to maintain a healthy and stable ecosystem. So, microbiomes with a lot of redundancy can keep doing important biological tasks and are better at dealing with changes in the environment. At this, the microbiome is really good because it has microbes that can do the same things which is called functional redundancy in the microbiome (Holzhausen et al., 2024). There is strong correlation between microbial community assembly and functional redundancy. Many of the microbial communities have services and means of coping with food and energy that ensures that the ecosystem remains healthy in spite of changing environmental conditions. Redundancy of microbial communities allows them to better cope with stress from living and non-living components can better transform food to nutrients, degrade pollutants, and even contribute to the health of the host. We now know a lot more about how microbial communities come and how the different species interact with each other in different environments thanks to new tools, like metagenomics, metabolomics and computer modeling which have really helped our understanding of microbial community assembly and functional redundancy (Chen et al., 2022).

### 4.2 Keystone taxa and functional guilds

Keystone taxa can be compared to vital support systems within microbial communities. Although they are present in relatively low abundance, they perform disproportionately important functions in sustaining community stability and ecological balance. These taxa significantly influence host health by regulating nutrient utilization and shaping interactions among microorganisms. When these “key” organisms are eliminated or decreased, the smaller living things in the community can suffer. Keystone taxa associated with plants can even help the plant to obtain nutrients to withstand disease, stress and proper growth. They do this by producing a substance(s) that is beneficial for the plants. It is very significant that keystone taxa have an important role in the health of the plants, and the community of living things that live with them (Banerjee et al., 2018). In a microbiome, functional guilds are similar to a team of living organisms that function together to perform the same role. It does not matter if they are very different from each other. It’s what they can do that is important. These little creatures are classified together because they share ability to perform similar functions such as fixing nitrogen, breaking down cellulose or clearing away substances in the environment. Functional guilds are of great importance in maintaining environmental stability. They ensure that key tasks are completed in environments. This is crucial, for keeping everything running smoothly. The functional guilds perform activities, such as degradation of pollutants, provisioning of phosphate to plants, and even methane and sulfur (Dasgupta et al., 2024). The interactions among keystone taxa and functional guilds are very significant for maintaining the functioning of the microbiome and balancing the environment. Even the microbes in the keystone are bossy. These interactions are implicated in nutrient availability, microbial succession, soil and plant disease resistance and environmental adaptation in soil and plant microbiomes. Recent advances in ecological functional guilds and keystone taxa identification, such as metagenomics, transcriptomics and network analysis (Xun et al., 2021), have significantly enhanced the characterization of microbial functional guilds in diverse ecosystems.

### 4.3 Microbiome stability under environmental stress

Microbial community structure is vulnerable to natural (e.g., drought, temperature, fire) and anthropogenic stressors (e.g., heavy metal exposure, land-use change). Despite the vast amount of data produced, there are no systematic cross-environmental analysis of microbiome responses to multiple disturbances (Rocca et al., 2019).

Environmental stress is rising globally, yet we still have no clear picture of how stress destabilizes the microbial communities and the ecosystem services they provide. We present the first evidence that naturally occurring microbiomes exhibit network properties indicative of unstable communities under persistent stress. One research show that by measuring changes in diversity and structure of soil microbiomes along 40 replicate stress gradients (elevation/water availability gradients) in the Florida scrub ecosystem: (1) prokaryotic and fungal diversity decline in high stress, and (2) two network properties of stable microbial communities—modularity and negative: positive cohesion—have a clear negative relationship with environmental stress, explaining 51–78% of their variation.

Environmental stressors occur at different magnitudes, frequencies and durations that introduce spatiotemporal heterogeneity in the environment. Spatiotemporal heterogeneity is a key driver both in the maintenance and depletion of biodiversity. In ecology, stressor, disturbance, perturbation, and threat are often used interchangeably and they refer to a variety of environmental changes (natural, anthropogenic, abiotic or biotic). Here, we refer to any factor that alters steady-state environmental conditions (biotic or abiotic) and impacts the growth or mortality of organisms in a community as a stressor, which may induce deterministic or stochastic changes in stationary relative abundance profiles of microbiomes (Hernandez et al., 2021).

Network analysis is a promising tool for understanding the effects of stress on the stability of microbial communities. Networks are mathematical representations of communities in ecology where nodes are individual taxa and edges are observed correlations in abundances among taxa, from which interactions can be inferred. Properties of networks (e.g., modularity, sparsity, etc.) have been successfully used to predict the stability of macroorganism networks such as plant-pollinator networks and food webs and have recently been applied to microbiomes (Etesami, 2024). Communities with certain network characteristics (higher modularity, lower positive associations among taxa, and higher negative associations among taxa) are especially stable, in the sense that these communities: (1) are less subject to shifts in composition in response to environmental perturbations and/or (2) are more likely to return to their equilibrium composition after a perturbation (Hernandez et al., 2021).

In the case of constant exposure of microbes to stressful environmental factors, network modularity and negative/positive cohesion play an important role in the resilience and stability of the community. In the case when a community possesses high network modularity, this means that there are species in such a community that form clusters and interact with one another. It helps to minimize the impact of disturbances on the network. This means that the network will be resilient in conditions of stressful environment. On the other hand, the interaction between negative and positive cohesion is indicative of the type of interaction in the microbial community. Negative cohesion occurs due to species competition that does not allow dominating and makes the community stable while positive cohesion occurs due to cooperation between species and provides for resource exchange (Hernandez et al., 2021).

## 5 Plant–microbiome interactions and host regulation

The interaction between plants and their microbiomes constitutes a dynamic and highly regulated process wherein plants have the capacity to influence the composition and function of their microbial communities, especially in the rhizosphere, phyllosphere, and endosphere. The plants produce a wide range of substances from their roots, which include sugars, amino acids, organic acids, and secondary metabolites. These serve as chemical signals and nutrient sources that recruit beneficial microbes while preventing the proliferation of pathogenic microbes (Berendsen et al., 2012). This selective recruitment results in the development of the core microbiome, which improves nutrient uptake and stress and disease resistance in plants. The interaction is further controlled by the plant’s immune system that differentiates between beneficial and pathogenic microbes using pattern recognition receptors (PRRs), which recognize MAMPs (Berendsen et al., 2012). Beneficial microbes can overcome or modulate the activation of plant immunity through the secretion of effector proteins, phytohormones, and volatile compounds that regulate the signaling pathways of the host plant associated with salicylic acid, jasmonic acid, and ethylene (Hacquard et al., 2017). Moreover, plants can genetically regulate the establishment of their microbiomes based on the traits coded within their genome, which affects the diversity and stability of their microbiome in varying environmental settings. The feedback regulation between plants and microbes is also significant, where microbial products can induce changes in plant gene expression, causing systematic variations in their physiological functions and defense mechanisms (Vandenkoornhuyse et al., 2015). Environmental variables such as the soil composition, climatic conditions, and agricultural methods have a significant impact on these interactions (Trivedi et al., 2020).

### 5.1 Functional redundancy in plant microbiomes: a key mechanism for ecosystem stability and resilience against environmental perturbations

The definition of functional redundancy in plant microbiome is the presence of multiple species within the plant microorganisms that have the capability of conducting similar ecological functions although being representatives of completely different taxonomical groups. In particular, some groups of microorganisms have similar metabolic pathways including nutrient cycles, nitrogen fixation, phosphate solubilization, production of phytohormones, disease resistance, and degradation of contaminants. Therefore, any extinction of some kinds of microorganisms will not cause the disruption of ecological process because other species are capable of performing similar functions (Ge, 2026).

The concept of microbial functional redundancy is regarded by researchers as one of the most effective ways of adapting to environmental conditions within a community of plants and microorganisms. In fact, such adverse impacts on the ecosystem as climate changes, shortage of water, increase of soil salinity, pollution, infections, as well as agricultural practices, may lead to extinction of certain groups of microorganisms. However, because of the redundancy of functions, new microorganisms will appear which will perform the following functions: provide plants with necessary nutrients, decompose organic materials, react to stress factors, and resist infections.

The existence of functional redundancy means that the processes which are beneficial to plants can still occur despite any changes that may be brought about by change in the makeup of the community in the rhizosphere or endosphere. Hence, it becomes assured that no matter what happens within the ecosystem, the plants will be healthy and productive (Shade, 2023).

### 5.2 Root exudates and microbial recruitment

The process of root exudation plays an important role in rhizosphere microbiome establishment through the supply of nutrients and signaling molecules involved in plant-microbe interactions. Root exudates include diverse combinations of small and macromolecules, such as carbohydrates, amino acids, organic acids, fatty acids, vitamins, enzymes, phenols, flavonoids, and various secondary metabolites, which can be secreted into soil via plant roots actively and passively (Bais et al., 2006). Plant species specificity, development, and environmental factors, like nutrient status, drought, and biotic stress, influence the synthesis and release of root exudates. Using this chemotactic response, plants attract beneficial microorganisms, e.g., PGPR and mycorrhizal fungi, and benefit from increased nutrient uptake (nitrogen fixation and phosphorus mobilization), stress resistance, and pathogen protection (Badri and Vivanco, 2009). For example, flavonoids released from legume roots function as important signaling molecules that trigger nod gene expression in rhizobia, leading to the establishment of nitrogen-fixing symbiosis, whereas strigolactones activate arbuscular mycorrhizal fungal spore germination and hyphal branching (Motavalli et al., 2004). Root exudates not only recruit microbes but can also modify microbial gene expression and activity to modulate phenotypes like chemotaxis, biofilm development, and quorum sensing. Similarly, plants can change their exudate profile when subjected to pathogenic attack to release antibacterial or antifungal compounds, or alternatively, signaling molecules that attract beneficial microbes that can inhibit pathogens, a process sometimes called the “cry for help” (Ma et al., 2022). The dynamic and mutualistic relationship creates a positive feedback cycle where microbes consume exudates and in return generate metabolites such as phytohormones, siderophores, and antibiotics that impact plant development and immune responses (Stringlis et al., 2018). Thus, root exudates play an important role in regulating the host microbiome to affect its architecture, diversity, and functionality (Rolfe et al., 2019).

### 5.3 Microbial modulation of plant detoxification pathways

**Figure 4:**
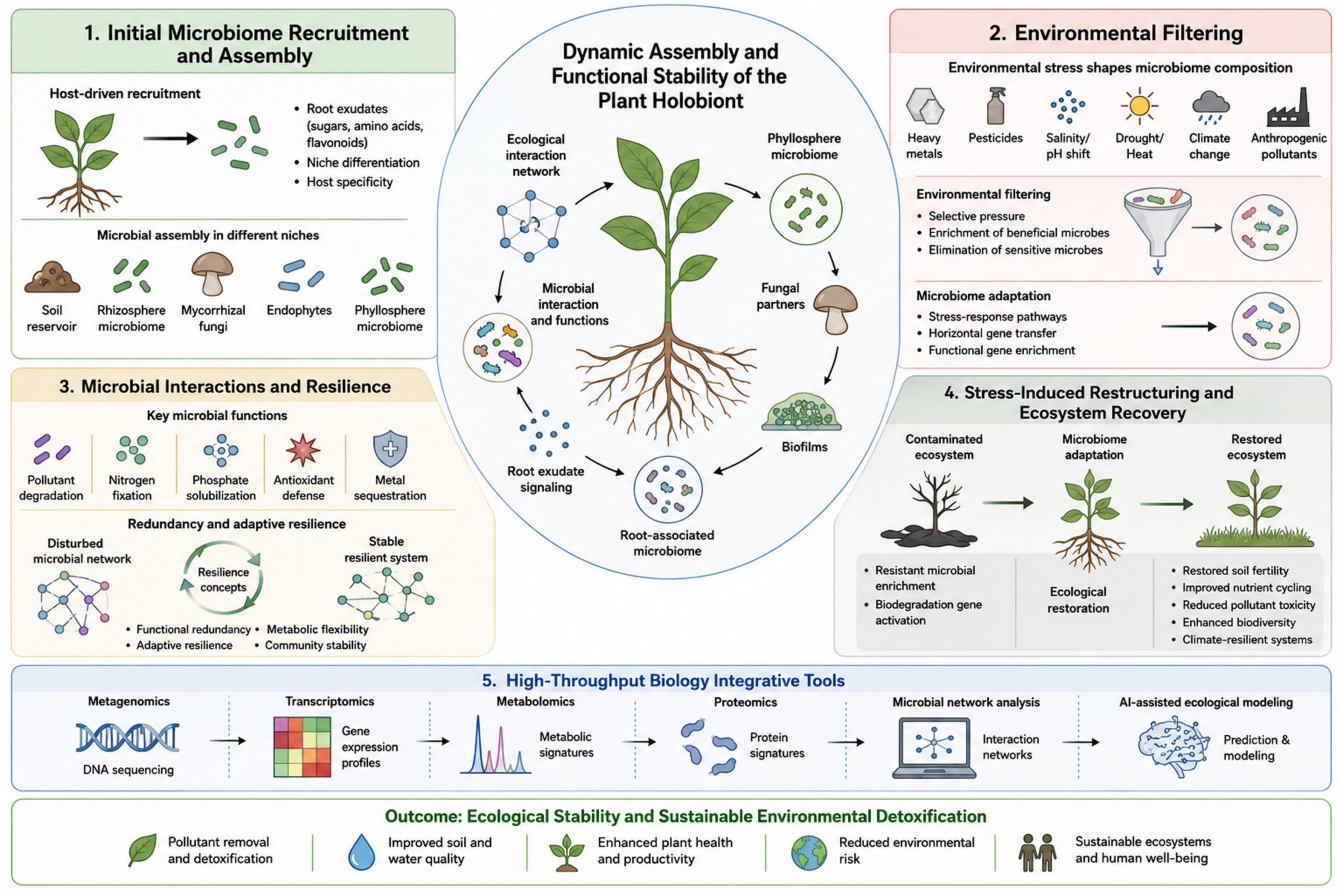
Ecological assembly, functional stability, and adaptive restructuring of plant microbiomes under environmental stress. The figure demonstrates how host-driven recruitment, environmental filtering, microbial interaction networks, functional redundancy, and adaptive resilience collectively shape microbiome assembly and stability under contaminated conditions. Dynamic restructuring of microbial communities promotes sustained ecosystem detoxification and ecological recovery.

The regulation of plant detoxification mechanisms by microbes is a crucial part of plant–microbiome interaction that allows plants to adapt to various pollutants and toxic substances present in their environment. Plant detoxification processes usually occur via three interlinked stages, which include phase I—transformation, phase II—conjugation, and phase III—sequestration of toxic substances with the participation of such important enzymatic groups as cytochrome P450 monooxygenases, GST, and UDP-glycosyltransferases. Plants can be affected in their detoxification process by beneficial bacteria inhabiting the rhizosphere or endosphere either directly or indirectly (Shaffique et al., 2022). Some PGPR and fungi have inherent metabolic potential for xenobiotic biodegradation or biotransformation of hazardous elements, pesticides, and other chemicals, thus limiting their bioavailability and toxicity for plants (Cao et al., 2023). Moreover, microorganisms can activate signaling mechanisms associated with stress and defense responses, resulting in enhanced gene expression in plants for detoxification purposes. These mechanisms include signaling by salicylic acid, jasmonic acid, and ethylene. In one study, it was revealed that the introduction of microorganisms increased the activity of glutathione S-transferase (GST) and antioxidant enzymes in plants under heavy metal stress, leading to enhanced elimination of reactive oxygen species (ROS) and ensuring homeostasis in cells. Endophytic microbes also synthesize secondary metabolites, such as siderophores, organic acids, and biosurfactants, which bind or immobilize the toxic elements, allowing for their compartmentalization in the host plant (Glick, 2012). Moreover, microorganisms synthesize VOCs and signaling molecules that prepare plants for detoxification in case of stress exposure (Gouda et al., 2018). In addition, this symbiosis process becomes more effective due to feedback loops, which involve chemicals produced by plants that affect the microbiome, thus making the detoxification potential of the holobiont optimal (Rizwan et al., 2015). The environmental factor, such as soil composition and type of pollutant, has a big impact on effectiveness. Generally, the microbe partners become additional elements of the plant metabolism, contributing to its ability to detoxify substances (Doty, 2008). To further explain how environmental stress and ecological interactions govern microbiome stability and adaptive restructuring, the ecological assembly dynamics of plant-associated microbial communities are illustrated in Figure 4.

### 5.4 Three interlinked biochemical phases (Phases I, II, and III) that plants undergo during detoxification

According to scientific research papers, the detoxification of plants against xenobiotics, which include pesticides, organic pollutants, and many others, is usually referred to as a three-stage “green liver” detoxification mechanism, similar to the detoxification performed by animal liver cells. Friendly bacteria in the soil around and within the roots are able to increase the effectiveness of all three phases of detoxification (Basapuram et al., 2025).

#### 5.4.1 Phase I: Transformation

During this phase, plants chemically modify substances through oxidation, reduction, or hydrolysis reactions. Functional groups such as hydroxyl, amino, or carboxyl groups are introduced, increasing the compound’s water solubility. This transformation is mediated by enzymes including cytochrome P450 monooxygenases, peroxidases, laccases, and esterases, marking the initiation of the plant detoxification process. In some cases, these reactions may generate more toxic intermediates that require further detoxification. Notably, the detoxification pathway in plants occurs in sequential stages and shares mechanistic similarities with detoxification processes observed in animals (Peng et al., 2021).

#### 5.4.2 Phase II: Conjugation

This stage involves the conversion of toxic intermediates produced during Phase I into conjugated compounds. Endogenous molecules such as glutathione, glucose, amino acids, and organic acids are attached to these compounds through conjugation reactions. This process is catalyzed by enzymes including glutathione S-transferases and glucosyltransferases. Conjugation increases the water solubility of toxic substances, facilitating their elimination from the body. Additionally, it reduces their toxicity and enhances their transport, making this phase essential for effective detoxification and waste removal (Peng et al., 2021).

#### 5.4.3 Phase III is sequestration and compartmentalization

In the final phase, conjugates are sequestered to vacuoles, cell walls, or lignin-like structures via ATP-binding cassette transporters (ABC transporters). The purpose of this phase is to isolate the toxic substances from normal metabolic processes and to protect plant cells from potential injury (Peng et al., 2021).

### 5.5 Beneficial endosphere or rhizosphere bacteria accelerate

Plant-associated microorganisms (PAMs), including plant growth-promoting rhizobacteria (PGPR) and endophytes, play a crucial role in enhancing plant growth and resilience. These microorganisms are also essential in assisting plants with pollutant detoxification. PAMs contribute to pollutant removal through multiple mechanisms, including the degradation of harmful chemicals before their uptake by plants. They produce detoxifying enzymes such as oxygenases and peroxidases, which help break down contaminants and reduce plant exposure to toxic compounds. Additionally, PAMs enhance phytoremediation by stimulating the activity of plant detoxification enzymes, thereby improving the overall efficiency of pollutant removal. Enzymes such as cytochrome P450 monooxygenases and glutathione S-transferases play essential roles in enabling plants to tolerate and detoxify harmful substances. Additionally, microorganisms in the rhizosphere significantly influence the bioavailability of pollutants through processes such as chelation and biodegradation, thereby facilitating pollutant uptake and removal by plants. These microorganisms also promote plant growth by enhancing root development through the production of bioactive compounds, including phytohormones and siderophores. Overall, plant-associated microorganisms are critical for both plant growth and efficient pollutant detoxification (Tariq and Farhat, 2025).

## 6 Translational approaches and microbiome engineering

Recent advances in plant microbiome engineering have created new opportunities to enhance phytoremediation efficiency in contaminated ecosystems. Unlike conventional remediation approaches that rely solely on plant physiology, microbiome engineering focuses on the deliberate manipulation of rhizospheric, endophytic, and phyllospheric microbial communities to improve pollutant degradation, heavy metal sequestration, and stress resilience. Strategies such as synthetic microbial consortia design, targeted bioaugmentation, host-guided microbiome selection, and microbiome-assisted gene editing are increasingly being explored to optimize detoxification outcomes. Engineered microbial communities can enhance xenobiotic degradation through specialized catabolic pathways, improve metal immobilization via biosorption and redox transformation, and strengthen plant tolerance to abiotic stress by modulating phytohormone signaling and antioxidant defenses. Furthermore, integrating multi-omics approaches, ecological modeling, and systems biology enables the identification of keystone taxa and functional guilds critical for microbiome stability and resilience under polluted conditions. These advances provide a framework for developing scalable, ecologically sustainable, and field-adaptable microbiome-based interventions for environmental restoration (Mimee et al., 2016). There are, however, some difficulties associated with creating safe, stable, and regulatory-compliant microbial consortiums as well as with accommodating individual differences between microbiomes (Charbonneau et al., 2020). Yet, with the help of such technologies as systems biology, computational modeling, and precision medicine, the efficiency and customization of microbiome-based interventions are likely to be improved, leading to clinical applications thereof (Sheth et al., 2019).

### 6.1 Microbial consortia and inoculation strategies

Microbial consortia and inoculation techniques are one of the key aspects of practical microbiome research, specifically in the fields of agriculture, environmental clean-up, and medical practice. Contrary to the approaches that make use of only a single strain of a microbe, microbial consortia rely on the mutual interactions between different types of microbes to boost stability and efficiency in various contexts (Trivedi et al., 2020). Microbial consortia tend to perform a range of coordinated functions, including nutrient cycling, biological control, and stress resistance (Niu et al., 2017). Microbial consortia design is facilitated by understanding the relationship between microbes, metabolic exchange, and ecological compatibility. Microbial inoculation techniques play an important role in the efficacy of these microbial consortia, with timing and mode of application being among those important elements that help achieve successful colonization (e.g., through seed coating, soil drenching, and foliar applications) (Zhang et al., 2009). Through advances in technology in terms of encapsulation and use of carriers among other techniques, the longevity and sustainability of microbes introduced into a given ecosystem have greatly been enhanced. Nonetheless, factors like competition with indigenous microorganisms, context dependence, and scale still pose major hurdles in the implementation of microbes. The application of microbial consortia in conjunction with precision agriculture practices and adaptive management is anticipated to improve their effectiveness (Grosskopf and Soyer, 2014).

### 6.2 Challenges in field-scale applications

**Figure 5:**
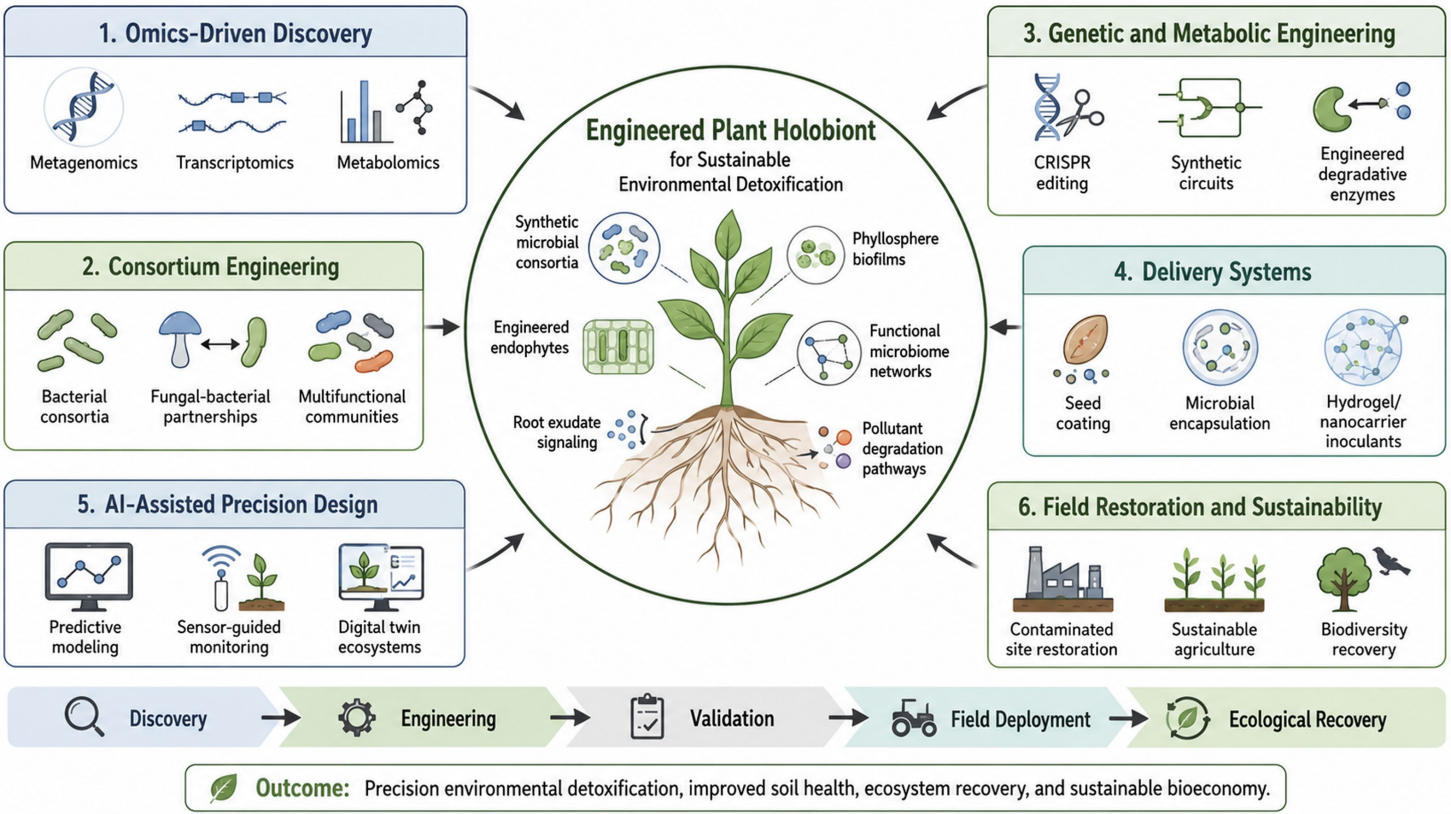
Engineering and translational strategies for plant microbiome-assisted environmental detoxification. The figure presents advanced approaches including omics-guided microbiome discovery, synthetic microbial consortium engineering, CRISPR-assisted genetic modification, smart delivery systems, and AI-assisted precision phytoremediation. These integrated translational strategies collectively support sustainable ecosystem restoration and next-generation environmental biotechnology.

The implementation of microbiome technologies at field scales presents many scientific and technical obstacles that usually result in failure to translate promising laboratory experiments into consistent and effective applications. The main problem that makes it difficult to obtain the anticipated results is the great variability and complexity of natural environments, which include variations in temperature, moisture levels, soil quality, as well as other factors affecting the ability of introduced microorganisms to survive, settle in the new environment, and perform their functions (Pellegrino et al., 2012). In most instances, the introduced strains are unable to take root because of competition with native microbiota and the presence of predators in the soil that inhibit the functioning of introduced organisms (Kaminsky et al., 2019). It is also important to note that there may be limitations regarding the prediction of ecological interactions and their long-term effects, leading to potential negative outcomes such as the disruption of the ecosystem of the native species or horizontal gene transfer (Malusá and Vassilev, 2014). There is an inadequacy in the regulatory system when considering the production of microbial products, which varies significantly from region to region and is insufficiently developed to cope with the advancements in synthetic biology (Sessitsch et al., 2018). Another major drawback is that there are no consistent protocols for the testing of microorganisms in field conditions and assessing their performance, making it difficult to accumulate reliable data for comparison between studies. To overcome these obstacles, researchers must adopt an integrated approach involving ecological concepts, complex modeling, and field validation over time, among other aspects (Vassilev et al., 2020). Emerging engineering and translational strategies aimed at enhancing microbiome-assisted environmental detoxification are comprehensively summarized in Figure 5.

## 7 Recent updated in various studies on plant microbiomes in environmental detoxification

Environmental detoxification research is advancing through the integration of multiple scientific disciplines, enabling a deeper understanding of microbiome functions within polluted ecosystems. Studies have shown that bad things like microplastics, pesticides, heavy metals and leftover medicines are really hurting the health of the soil the variety of microbes and the stability of plants (Abideen et al., 2022; Tang et al., 2024; Tanveer et al., 2024). This means we really need to find ways to clean up the environment that’re good for the earth. Other studies have found that when we are around these pollutants it can cause health problems like inflammation problems with how our bodies work and breathing issues. This shows that pollution is not just bad for the environment it is also bad for our health. Researchers have been looking at how microbiomes work. They found out that the community of microbes is shaped by what the host needs the pressure from pollutants and how the microbes fit into their environment (Ali et al., 2022; Berendsen et al., 2012; Etesami, 2024; Khan, 2023; Mimee et al., 2016). All of these studies show that microbiomes are really important for keeping ecosystems healthy when they are under stress from toxins. At the time other studies have found that groups of microbes work together to clean up bad things in both plants and animals. They can change arsenic into something harmful break down toxic substances and change toxins into something less bad. This means that microbiomes can take things and make them less harmful which helps the host and the environment get better (Berendsen et al., 2012; Compant et al., 2019; Patyal et al., 2025; Trivedi et al., 2020). All of these types of research presented in Table 4 show that microbiomes associated with plants are not just along for the ride they are actually helping to clean up the environment restore ecosystems and keep the environment healthy for a long time. Environmental detoxification research and microbiomes are really important for ecosystem restoration and environmental sustainability. Microbiomes play a crucial role in environmental detoxification, and a deeper understanding of their functions is essential for maintaining ecosystem health.

**Table 4:** Comparative overview of major research study designs used in environmental detoxification and plant microbiome investigations.

Study type	Primary objective	Major findings/outcomes	Relevance to plant microbiome-based environmental detoxification	References
Systematic analysis	To evaluate the environmental fate and ecotoxicological effects of micro- and nanoplastics	Demonstrated that microplastics accumulate across terrestrial and aquatic ecosystems and induce oxidative stress, inflammatory responses, and microbial dysbiosis	Supports the need for microbiome-assisted biodegradation and ecological restoration strategies	Abideen et al. (2022)
	To assess human health impacts associated with environmental microplastic contamination	Identified major exposure pathways and highlighted systemic toxicity linked to long-term plastic exposure	Emphasizes the importance of biological detoxification systems capable of reducing pollutant bioavailability	Vasse and Melgert (2024), Sohail et al. (2023)
	To investigate ecological impacts of microplastics on soil–plant systems	Reported disruption of nutrient cycling, plant growth inhibition, and altered rhizospheric microbial diversity	Establishes the ecological importance of rhizosphere microbiomes in restoring contaminated soils	Allouzi et al. (2021), Thijs et al. (2016)
	To examine natural microbial detoxification of antibiotics in environmental systems	Revealed that environmental microbiota possesses adaptive mechanisms for antibiotic degradation and resistance regulation	Highlights microbial functional plasticity and biodegradation potential under pollutant stress	Baquero et al. (2022), Thijs et al. (2016)
	To critically evaluate organophosphorus pesticide toxicity and bioremediation approaches	Demonstrated that microbial enzymatic pathways significantly enhance pesticide detoxification efficiency	Reinforces the role of microbial consortia in sustainable xenobiotic degradation	Tanveer et al. (2024), Albureikan (2023)
Meta-analysis	To synthesize evidence linking plastic-associated chemicals with human health disorders	Confirmed significant associations between plastic exposure and endocrine, metabolic, and inflammatory diseases	Provides epidemiological support for urgent development of eco-friendly detoxification technologies	Kour et al. (2021)
	To assess the association between microplastics and inflammatory bowel disease	Reported strong correlations between plastic-induced gut dysbiosis and chronic intestinal inflammation	Demonstrates how microbiome disruption contributes to pollutant-mediated disease mechanisms	Agrawal et al. (2024)
	To evaluate food-borne exposure routes of micro- and nanoplastics	Identified dietary intake as a major pathway for chronic contaminant exposure	Highlights the need for agricultural microbiome interventions to reduce contaminant transfer into food systems	Kumar et al. (2024), Meng et al. (2025)
	To investigate respiratory consequences of plastic pollution exposure	Found associations between airborne plastic particles and chronic respiratory dysfunction	Supports atmospheric detoxification roles of phyllosphere-associated microbiomes	Vasse and Melgert (2024)
	To summarize environmental and public health implications of plastic-associated pollutants	Demonstrated broad ecological and legal concerns regarding plastic contamination and human exposure	Reinforces the urgency for integrated ecological remediation strategies	Belmaker et al. (2024), Rocca et al. (2019)
Statistical analysis	To statistically evaluate factors controlling Pb accumulation in plants	Identified soil biogeochemistry and microbial community composition as major predictors of metal uptake	Demonstrates how microbial ecology influences heavy metal phytoremediation efficiency	Albureikan (2023), Tariq and Farhat (2025)
	To analyze microbiome assembly and network complexity across crop systems	Revealed that host selection strongly shapes microbial diversity and ecological interactions	Supports the concept of host-directed microbiome engineering for detoxification	Li S. et al. (2022), Xiong et al. (2021b), Yue et al. (2024)
	To investigate bacterial community assembly across plant niche compartments	Identified ecological filtering and niche specialization as drivers of microbiome organization	Highlights ecological compartmentalization of detoxification-associated microbes	Liu et al. (2021), Moroenyane et al. (2021), Yuan et al. (2022)
	To examine microbial assembly processes under Cd and Zn stress	Demonstrated pollutant-induced restructuring of microbial co-occurrence networks	Indicates adaptive resilience of microbiomes in metal-contaminated ecosystems	Xing et al. (2023), Jing et al. (2025), Thanwisai et al. (2022)
	To determine how plant developmental stages influence microbiome assembly	Showed dynamic shifts in microbial balance between deterministic and neutral ecological processes	Provides ecological insights into temporal stability of detoxification-associated microbiomes	Albureikan (2023), Moroenyane et al. (2021), Xiong et al. (2021a)
Cohort study	To evaluate cooperative microbiome-mediated arsenic detoxification in agricultural soils	Identified microbial consortia involved in arsenate reduction and arsenic methylation pathways	Demonstrates cooperative detoxification mechanisms in contaminated agricultural ecosystems	Rueangmongkolrat et al. (2024)
	To assess microbiome-assisted mitigation of arsenic toxicity in crops	Reported enhanced plant tolerance and reduced arsenic accumulation through beneficial microbiomes	Supports the use of microbiome engineering in sustainable agriculture	Ali et al. (2022), Tariq and Farhat (2025)
	To investigate gut microbial detoxification of plant toxins in herbivores	Demonstrated that gut bacteria actively metabolize toxic phytochemicals improving host adaptation	Expands understanding of microbiome-driven detoxification across biological systems	Tan et al. (2024), Zhang et al. (2024)
	To study glucosylation-mediated detoxification of plant toxins by silkworm microbiota	Identified microbial metabolic pathways involved in detoxification and xenobiotic tolerance	Highlights conserved microbial detoxification strategies relevant to plant systems	Yuan et al. (2023)
	To examine microbiota-mediated detoxification and defense suppression in insect systems	Revealed microbial contribution to toxin resistance and host defense modulation	Suggests ecological parallels between insect-associated and plant-associated detoxification microbiomes	Coolen et al. (2024), Kline and Joshi (2024), ul Malook et al. (2025)

## 8 Future perspectives and research gaps

### 8.1 Bridging microbial ecology and applied detoxification

Even though substantial progress has been made toward understanding plant microbiomes and their role in the detoxification process within the environment, there still exist many scientific and technical constraints which prevent researchers from developing reliable microbiome-based techniques for detoxification under field conditions (Simmer and Schnoor, 2022; Thijs et al., 2016). The past research on the use of microbiomes for detoxification has largely focused on individual microbes, specific catabolic processes, or experiments conducted within laboratories without considering such concepts as ecological interaction, functional redundancy, and ecosystem stability (Compant et al., 2019; Trivedi et al., 2020). Hence, in order to understand the interplay between microbiome structure, its dynamics, and ecosystem stability for efficient detoxification, it is necessary to conduct further research using a systems approach which encompasses various subdisciplines, microbial ecology, environmental microbiology, systems biology, and ecological engineering (Fadiji et al., 2025, Li H. et al., 2025).

Plant-mediated recruitment of beneficial microbes in stressful environments is a very important area that requires much attention (Rolli et al., 2021). Root exudates consist of carbohydrates, amino acids, flavonoids, coumarins, organic acids, and phytohormones and play a key role in the formation of the rhizosphere community and recruitment of beneficial microbes (Molefe et al., 2023). In line with the “cry-for-help” concept, plants exposed to pollutants selectively recruit microbes that can reduce toxic effects on plants and help them cope with stress (Rolli et al., 2021). Often, such microbes perform multiple functions for promoting plant growth by solubilizing phosphates, producing siderophores, possessing ACC deaminase, fixing nitrogen, and synthesizing phytohormones (Khatoon et al., 2024; Tariq and Farhat, 2025). However, molecular signaling mechanisms that control communication between plants and microbes require additional study (Molefe et al., 2023).

The assembly and stability of microbiomes in the presence of contaminants have not been explored well (Trivedi et al., 2020). The plant-microbiome systems represent very complex ecosystems that are controlled by host genetics, soil types, contaminant nature, weather, and competition among microorganisms (Li H. et al., 2025). Ecological selection, based on root secretion and environmental stress, favors degradation and stress-resistant microbes that can tolerate contaminant stress (Thijs et al., 2016). Nonetheless, these ecological mechanisms are highly affected by drought, salinity, high temperature, and other environmental disturbances (Trivedi et al., 2020). Therefore, ecological research is important for evaluating the resistance and successional dynamics of microbiomes during ecosystem restoration (Simmer and Schnoor, 2022).

Polluted and harsh environments may serve as sources of microorganisms possessing unique detoxification abilities and stress tolerance (Biswas et al., 2024). *Pantoea tagorei* sp. nov. is one example of bacteria isolated from the rhizosphere soil of coal mines, which have the ability to dissolve potassium, phosphate, fix nitrogen, and adapt to unfavorable environmental conditions (Biswas et al., 2024). Likewise, rhizosphere and endophytic bacteria isolated from metal-polluted soils often show adaptive features related to oxidative stress tolerance, siderophore production, and effective rhizosphere colonization (Khatoon et al., 2024). Therefore, these microorganisms can be regarded as promising candidates for future microbiome-assisted bioremediation applications.

Some recent studies have provided further insight into the microbial processes related to detoxification against heavy metals (Li H. et al., 2025; Tariq and Farhat, 2025). The detoxification process of heavy-metal-resistant PGPMs through biosorption, siderophores, the formation of organic acids, EPS formation, cellular uptake of metals, the production of antioxidants, efflux pumps, and redox reactions of enzymes takes place (Tariq and Farhat, 2025). The simultaneous reduction of toxic effects of metals, nutrient availability, and stress resistance of plants takes place (Khatoon et al., 2024). In addition, microbial synergism through consortia has increased the efficiency of detoxification due to metabolic interactions and sequential degradations (Kour et al., 2021).

One major challenge in modern remediation studies is the unreliable performance of inoculated microorganisms in the field environment (Simmer and Schnoor, 2022). Despite favorable results recorded in laboratory tests, there have been instances where the inoculated microorganisms do not successfully thrive within their new environment because of factors such as environmental changes, ecological competitions, and reactions between the introduced microorganisms and those present in nature (Compant et al., 2019). Thus, there has been an increasing focus on developing SynComs and microbial consortia engineered through the use of ecological cooperation, functional redundancy, and adaptability (Li H. et al., 2025; Trivedi et al., 2020). Unlike inoculation with monoculture microorganisms, the development of SynComs focuses on mimicking the behavior of natural microbial communities (Trivedi et al., 2020).

Environmental detoxification represents another important area of investigation when discussing synthetic biology and microbiome engineering (Li H. et al., 2025). Improvements in genome engineering techniques based on CRISPR, genome-wide metabolic models, and programmable microbes could contribute toward generating microorganisms that would be capable of acquiring additional catabolic capacities and stress resistance (Li H. et al., 2025). However, there are still major problems related to biosafety, ecological impacts, horizontal gene transfer, and regulation that remain a barrier to the use of engineered microorganisms in the environment (Simmer and Schnoor, 2022).

**Figure 6:**
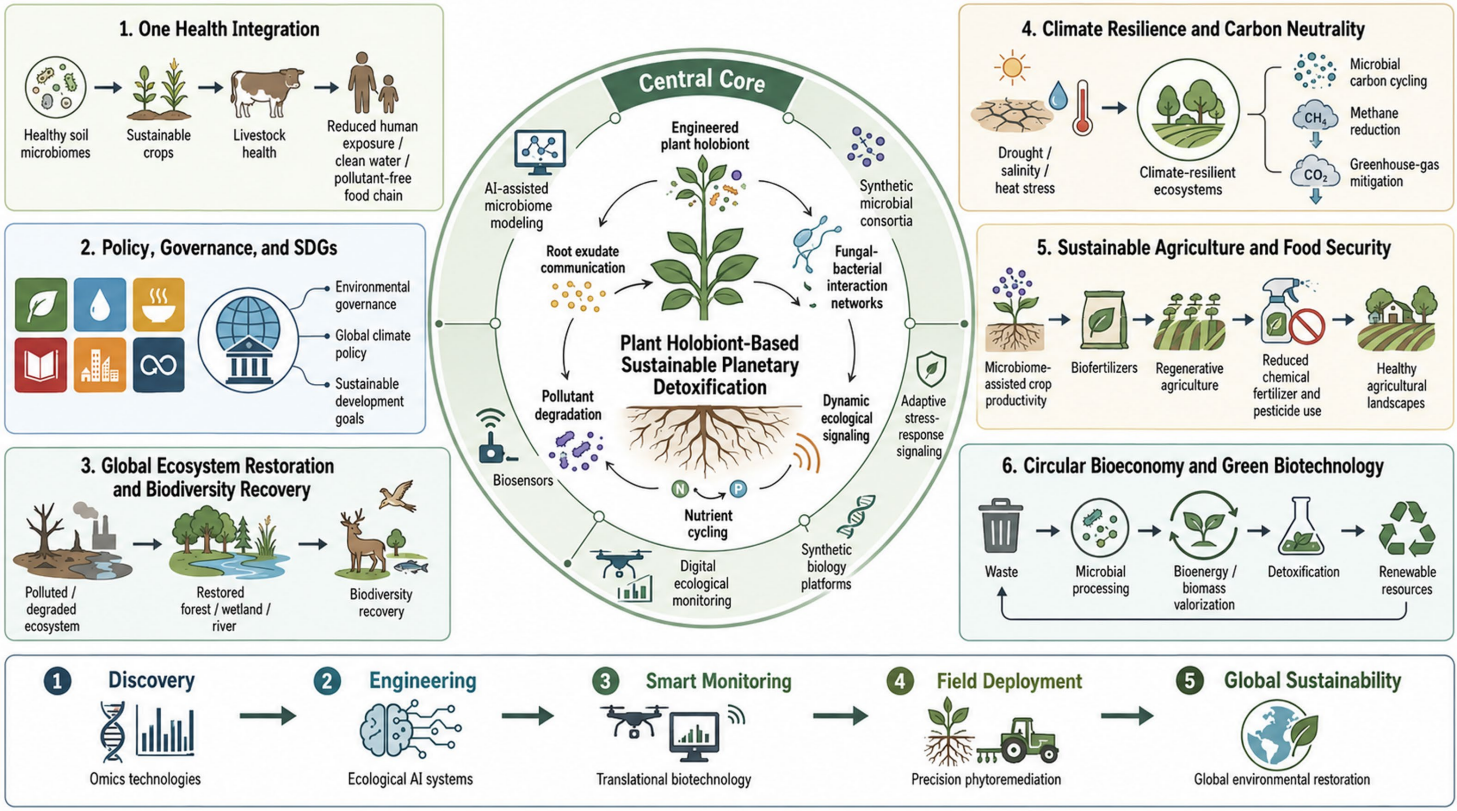
Future perspectives and integrated sustainability framework of plant microbiome-mediated environmental detoxification. The figure highlights the future role of engineered plant holobionts in supporting One Health integration, climate resilience, sustainable agriculture, circular bioeconomy development, precision microbiome engineering, biodiversity restoration, and global environmental sustainability. Interdisciplinary integration of ecological biotechnology and systems biology offers transformative opportunities for sustainable planetary health management.

A further evolving approach consists of linking up microbial treatment technologies with functional materials and bioelectrochemical approaches (Chojnacka et al., 2023). The functional materials, including biochar, activated carbon, zeolite, and nanomaterials, can act as carriers for microorganisms and pollutants to improve microbial survival and detoxification (Chojnacka et al., 2023). Chojnacka et al. (2023) introduced the idea of “plant-microorganism-functional material triangle” with respect to synergistic roles of plants, microorganisms, and functional materials in rhizoremediation. The interaction of the three factors is referred to as the plant-microorganisms-functional materials triangle, and this can be described as a complex process of phytoremediation. In such systems, plants provide microbial life with root exudates and microhabitats, whereas microorganisms play an important part in plant growth and pollutant degradation. The primary function that functional materials such as biochar, activated carbon, zeolites, and nanomaterials perform is that of catalyzers as they aid in the growth of both plants and microorganisms, thus making the process of phytoremediation more efficient. These materials have the capacity to remove pollutants from the environment and to make microbial biofilms. Similarly, the application of extracellular electron transfer mechanism through bioelectrochemical systems might facilitate detoxification of toxicants like chromium and mercury (Li H. et al., 2025). It is expected that Artificial intelligence (AI), machine learning, and predictive ecology will influence microbiome research in the future (Fadiji et al., 2025). Predictions for microbial community dynamics can be improved through ecological network and omics data integrated into AI algorithms, which have potential to enhance understanding about remediation efficacy (Fadiji et al., 2025; Li H. et al., 2025). Predictive ecology models will thus facilitate the use of microbial communities in environmental remediation based on climate changes. The future translational potential and global sustainability implications of plant microbiome-mediated detoxification within a One Health framework are conceptually summarized in Figure 6.

### 8.2 Translational strategies for sustainable microbiome-based environmental detoxification

Strategies for future environmental detoxification should consider aspects like sustainability, resilience toward climate change, and restoration of ecosystems instead of focusing on just removing contaminants in the short term (Kour et al., 2021). Technologies that make use of the microbiome of plants for the purpose of remediation can be considered environmentally friendly alternatives to traditional physicochemical technologies, which tend to be associated with high operational costs, secondary contamination, and disturbance of soil ecological balance (Simmer and Schnoor, 2022). Biological methods such as phytoremediation, rhizoremediation, bioaugmentation, biostimulation, and microbiome-based agriculture are becoming increasingly popular (Compant et al., 2019).

It is expected that microbial consortia will be playing crucial roles in the detoxification processes of the future, due to the fact that cooperation among microbes during their metabolic processes can lead to improved degradation of pollutants compared to what can be achieved using only one strain (Kour et al., 2021; Li H. et al., 2025). The metabolites produced from one microbial species act as substrates for the other, hence enhancing the process of degradation and making them more adaptive and resilient against environmental stresses (Kour et al., 2021). This is particularly important when dealing with the degradation of organic compounds and contaminant systems with multiple pathways necessary for degradation (Li H. et al., 2025).

Sustainable detoxification systems must also take into account the use of native microorganisms adapted to the specific local environment (Compant et al., 2019). Native microorganisms tend to be more ecologically adaptable and resistant to stress as compared to foreign inoculants (Simmer and Schnoor, 2022). Studies of rhizospheric microorganisms extracted from polluted soils have revealed the presence of numerous functional abilities in these organisms, ranging from the ability to dissolve phosphates, fix atmospheric nitrogen, produce siderophores, exhibit osmo-protection, and resist heavy metals (Biswas et al., 2024, Khatoon et al., 2024; Tariq and Farhat, 2025).

Sustainable remediation technologies need to increasingly consider how microbiome-assisted farming can be combined with ecological restoration and soil health (Compant et al., 2019; Trivedi et al., 2020). Microbes not only assist with pollutant removal but have an important role to play in nutrient recycling, soil structure, drought resistance, pathogen reduction, and climate change resistance (Khatoon et al., 2024; Molefe et al., 2023). Ecological functions such as these mean that microbiome-assisted remediation is especially suitable for degraded farmlands, mining and industry sites (Li H. et al., 2025).

The use of remediation techniques combined with functional materials and green technology represents one of the most attractive directions for the future (Chojnacka et al., 2023). The use of biochar, activated carbon, zeolites, clay minerals, and nanomaterials can help improve the efficiency of pollution remediation because these materials will act as pollutants’ adsorbents, microbial cells carriers, and electron donors (Chojnacka et al., 2023). Similarly, microbiome-based technologies coupled with bioelectrochemical systems can help improve the efficiency of electron exchange during reduction/oxidation processes of pollutants (Li H. et al., 2025).

Host plant selection and microbiome-enabled breeding initiatives are expected to contribute significantly to future sustainable remediation systems (Trivedi et al., 2020). Genotype has a pronounced impact on root exudate production, microbe attraction, and rhizosphere formation under stress from pollutants (Molefe et al., 2023). Hence, the future breeding approach may involve cultivating host plants that can recruit degrading microbes selectively and sustain ecological relationships amid harsh environmental stresses (Rolli et al., 2021). The holobiont paradigm, which includes both the plant and its microbiome as an ecological unit, is expected to be a key element in sustainable environmental stewardship and resilient agriculture (Compant et al., 2019).

Finally, long-term ecological monitoring, biosafety assessment, regulatory standardization, and interdisciplinary collaboration will be essential for successful implementation of microbiome-based detoxification technologies. Integration of expertise from microbiology, ecology, environmental engineering, agronomy, computational biology, and systems biology will be necessary to translate microbiome research into scalable and socially acceptable remediation strategies. Long-term ecological monitoring, biosafety evaluations, standardization of regulations, and interdisciplinary cooperation are critical for implementing microbiome-detoxification technologies effectively (Fadiji et al., 2025; Simmer and Schnoor, 2022). It will be important to integrate expertise in microbiology, ecology, environmental engineering, agriculture, bioinformatics, and systems biology to enable successful implementation of microbiome research in large-scale, socially acceptable systems for environmental detoxification (Fadiji et al., 2025). In summary, the future development of environmentally sustainable detoxification strategies is expected to transition away from classic mono-specific technologies toward a more complex and ecologically oriented microbiome engineering approach (Li H. et al., 2025; Trivedi et al., 2020).

The possibility of successful application of any promising microbial inoculants obtained from laboratories in field environments does not automatically follow due to the complexities and unpredictability of field conditions. Factors associated with the nature of the soil, weather, nutrition, plant, competition by the natural community of microbes, environmental stress, and other agricultural activities can affect the effectiveness of the inoculants used. Although experiments conducted in laboratories have been done under controlled conditions, natural microbes present in the field environment can inhibit the introduction of the inoculants.

### 8.3 Limitations of using modern multi-omics data

Multi-omics methods, like metagenomics, metatranscriptomics, metaproteomics, and metabolomics, have immense potential for yielding critical information on the soil microbiome profile in the case of environmental contamination; however, several computational and biological hurdles can affect their performance. Firstly, regarding computational issues, multi-omics involves processing large volumes of data requiring high memory capacity, computing powers, bioinformatics software applications, and statistical analysis skills. In terms of the latter, some of the major computational obstacles include incomplete database used for comparison purposes, genome assembly and annotation, integrating multi-omics datasets, and separation of signal from noise. On the biological level, the main limitation of multi-omics lies in the huge variety of microbial species existing in the soil ecosystem. There are many uncultivable microorganisms in the soil with little annotated genomes. Moreover, the simple existence of various forms of nucleic acids, proteins, and metabolites may not imply the actual presence of active microorganisms. Due to the great heterogeneity of soil ecosystems, microbial communities, and contaminants, it becomes challenging to describe the soil’s microbiome properly (Li et al., 2026).

## 9 Conclusion

Plant microbiomes play a vital role in environmental detoxification and ecosystem resilience. Their effectiveness extends beyond the host plant’s metabolic capacity and relies on the diverse microbial communities inhabiting the rhizosphere, endosphere, and phyllosphere. These microorganisms contribute to pollutant degradation, metal transformation, redox regulation, antioxidant defense, and nutrient cycling, thereby enhancing plant survival and adaptation in contaminated environments.

Environmental decontamination processes are regulated by intricate interactions between plants, microbes, and their environment. Microbial community establishment, functional redundancy, keystone species interactions, and microbial colonization by plants play critical roles in determining remediation success and maintaining ecological integrity. Furthermore, root exudate signaling and microbial modulation of plant detoxification pathways exemplify the high level of coordination between plants and microbes in times of environmental stress. Moreover, due to their versatile functions, plant-microbe relationships can simultaneously achieve contaminant degradation, plant growth stimulation, nutrient recycling, and resistance to stress.

Recent developments in omics-based research, ecological network studies, synthetic biology, and microbiome engineering have considerably advanced knowledge about microbial functional ecology and led to the development of future-oriented bioremediation methods. New methods like synthetic microbial communities, microbiome-enhanced phytoremediation, bioelectrochemical systems, and combining functional materials like biochar and nanocarriers could enhance the effectiveness and applicability of bioremediation systems. Nonetheless, problems like ecological variation, unpredicted field conditions, safety issues, effects of climate change, and new contaminants remain to be investigated further.

This review represents a holistic approach toward the ecological and functional aspects of plant microbiomes for detoxification purposes, hence providing the basis for future studies in the field. An in-depth comprehension of ecology and functional relations between plants and their microbial communities would be indispensable for the development of sustainable climate-change resistant bioremediation tools applicable in the real world.
